# Roles of Macrophages in Atherogenesis

**DOI:** 10.3389/fphar.2021.785220

**Published:** 2021-11-26

**Authors:** Lia Farahi, Satyesh K. Sinha, Aldons J. Lusis

**Affiliations:** ^1^ Monoclonal Antibody Research Center, Avicenna Research Institute, Tehran, Iran; ^2^ Department of Medicine, David Geffen School of Medicine, University of California, Los Angeles, Los Angeles, CA, United States

**Keywords:** atherosclerosis, inflammation, macrophages, lipid, plaque, biomarker

## Abstract

Atherosclerosis is a chronic inflammatory disease that may ultimately lead to local proteolysis, plaque rupture, and thrombotic vascular disease, resulting in myocardial infarction, stroke, and sudden cardiac death. Circulating monocytes are recruited to the arterial wall in response to inflammatory insults and differentiate into macrophages which make a critical contribution to tissue damage, wound healing, and also regression of atherosclerotic lesions. Within plaques, macrophages take up aggregated lipoproteins which have entered the vessel wall to give rise to cholesterol-engorged foam cells. Also, the macrophage phenotype is influenced by various stimuli which affect their polarization, efferocytosis, proliferation, and apoptosis. The heterogeneity of macrophages in lesions has recently been addressed by single-cell sequencing techniques. This article reviews recent advances regarding the roles of macrophages in different stages of disease pathogenesis from initiation to advanced atherosclerosis. Macrophage-based therapies for atherosclerosis management are also described.

## 1 Introduction

**FIGURE 1:**
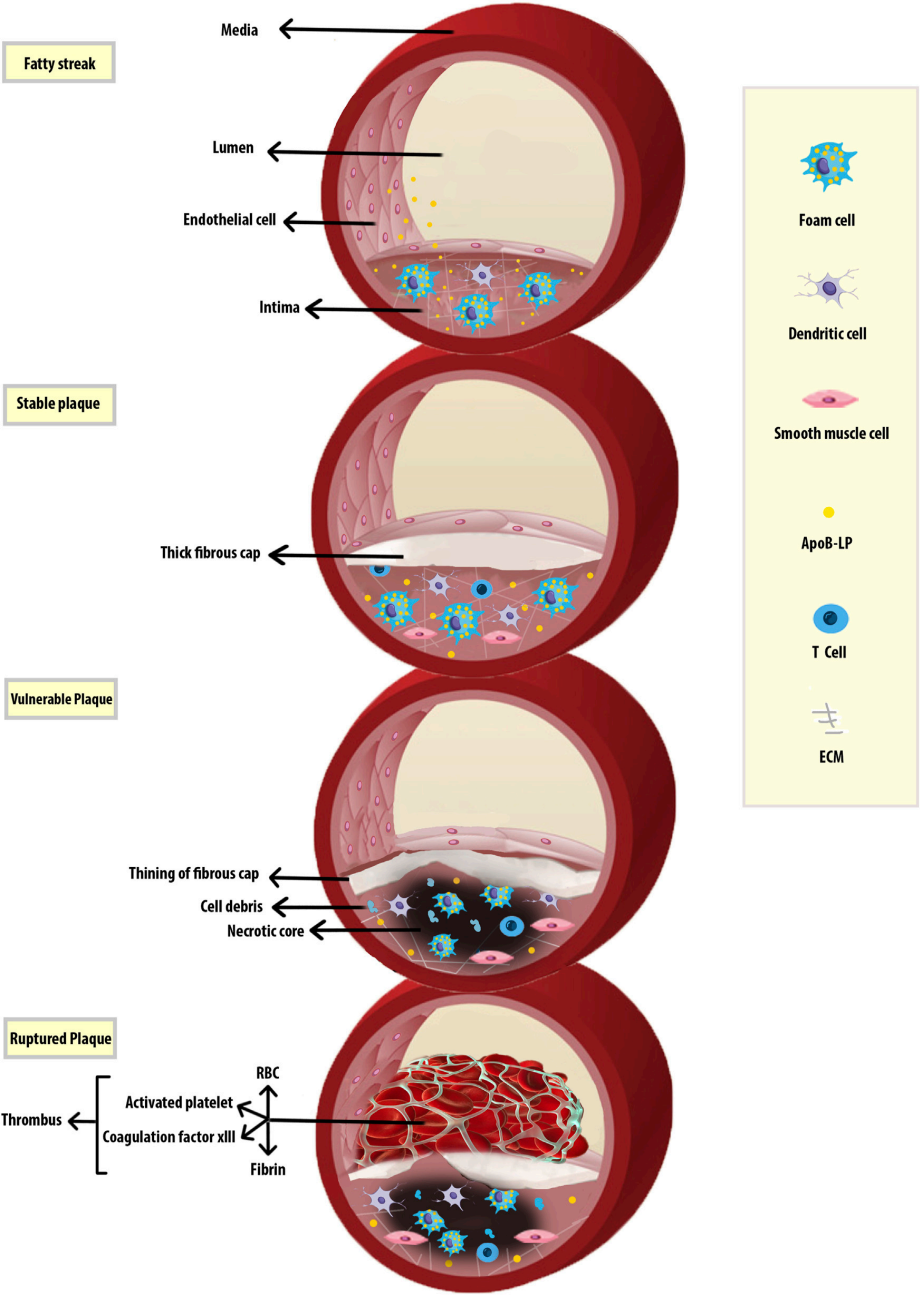
Progression of Atherosclerosis. Early fatty streak lesions are characterized by subendothelial accumulation of ApoB-LPs, the main protein constituent of lipoproteins such as VLDL and LDL which promote the recruitment of Mφs. As the atherosclerotic lesion progresses, ApoB-LP retention is amplified. Vulnerable plaques are characterized by the accumulation of ACs and defective efferocytosis, resulting in the lipid-filled necrotic core. A thinning fibrous cap reduces plaque stability and makes them susceptible to rupture and thrombus formation. Apolipoprotein B-containing lipoproteins (ApoB-LPs).

Atherosclerosis (AS) is associated with both metabolic dysfunction and chronic inflammatory processes. AS is initiated by endothelial dysfunction caused by factors such as high plasma cholesterol, hypertension, diabetes, leukocytosis, cigarette smoking, and low shear stress (Xu et al., 2019). A particularly central event is the subendothelial accumulation of low-density lipoproteins (LDL) and very-low-density lipoproteins (VLDL), which are then subjected to aggregation, oxidation, and enzymatic modifications. The resulting oxidized phospholipids (OxPLs), then contribute to inflammatory processes that promote the activation of endothelial cells (ECs) to express several types of leukocyte adhesion molecules (Marchio et al., 2019). This, in turn, results in leukocyte recruitment, including blood monocytes, neutrophils, lymphocytes, and also platelets to the vessel wall. The monocytes differentiate into macrophages (Mφs) which then proliferate in response to Mφ colony-stimulating factor (M-CSF) and other factors (Robbins et al., 2013b; Sinha et al., 2021). Local proliferation dominates lesional Mφ accumulation in AS. Monocyte recruitment can not fully account for lesional Mφ accumulation in established AS and lesional Mφ can replenish either through the continuous recruitment of circulating monocytes or through some other processes. Mφ self-renewal was identified as a therapeutic target for cardiovascular diseases (Robbins et al., 2013a). Mφs can polarize and reprogram their functional phenotypes in response to different stimuli, and thus display distinct functions related to tissue homeostasis and inflammation (Park, 2021). Mφs take up the aggregated and oxidized LDL (Ox-LDL) leading to their transformation into cholesterol engorged foam cells. Such uptake of modified lipoproteins is mediated primarily by scavenger receptors (SRs) expressed on Mφs. Besides Mφs, vascular smooth muscle cells (VSMCs) can also transdifferentiate into lesional Mφ-like cells through cholesterol loading (Rong et al., 2003). Advanced plaques are characterized by subendothelial deposition of lipids, necrotic cell debris, calcium phosphate crystals, fibrosis, and inflammatory immune cells including Mφs, T and B lymphocytes, neutrophils, dendritic cells (Canet-Soulas et al., 2021). Lesional T cells can recognize local antigens, leading to activation, clonal expansion, and cytokine production (Robertson and Hansson, 2006). Leukocytosis, an increase in the numbers of circulating leukocytes, is associated with cardiovascular (CV) diseases. Some leukocytes are atherogenic and sustain oxidative stress (OS) and inflammation after myocardial infarction (MI) while others are atheroprotective and help it resolve (Swirski and Nahrendorf, 2013b). For example, CD4^+^ T cell deficiency results in a delay in the switch of Ly6C^hi^ into Ly6C^Low^ monocytes and impaired healing. The inflammatory Ly-6C^hi^ phenotypes are required during the early response to ischemic injury, but if they persist in the infarct too long, the reparative functions of Ly-6C^low^ monocytes are impaired (Nahrendorf et al., 2007; Leuschner et al., 2012). The progression of an atherosclerotic lesion including the formation of early fatty streak lesions, fibrous cap, necrotic core, and thrombus have been displayed in Figure 1 (Virmani et al., 2002; Chinetti-Gbaguidi et al., 2015). In this review, we provide an overview of the critical role of Mφs in the pathogenesis of AS, including recent findings relating to clonal hematopoiesis, single-cell sequencing, and senescence. Mφ-based therapies for the management of AS are also discussed.

## Monocyte Adhesion to the Endothelium and Entry Into the Subendothelial Space

**FIGURE 2:**
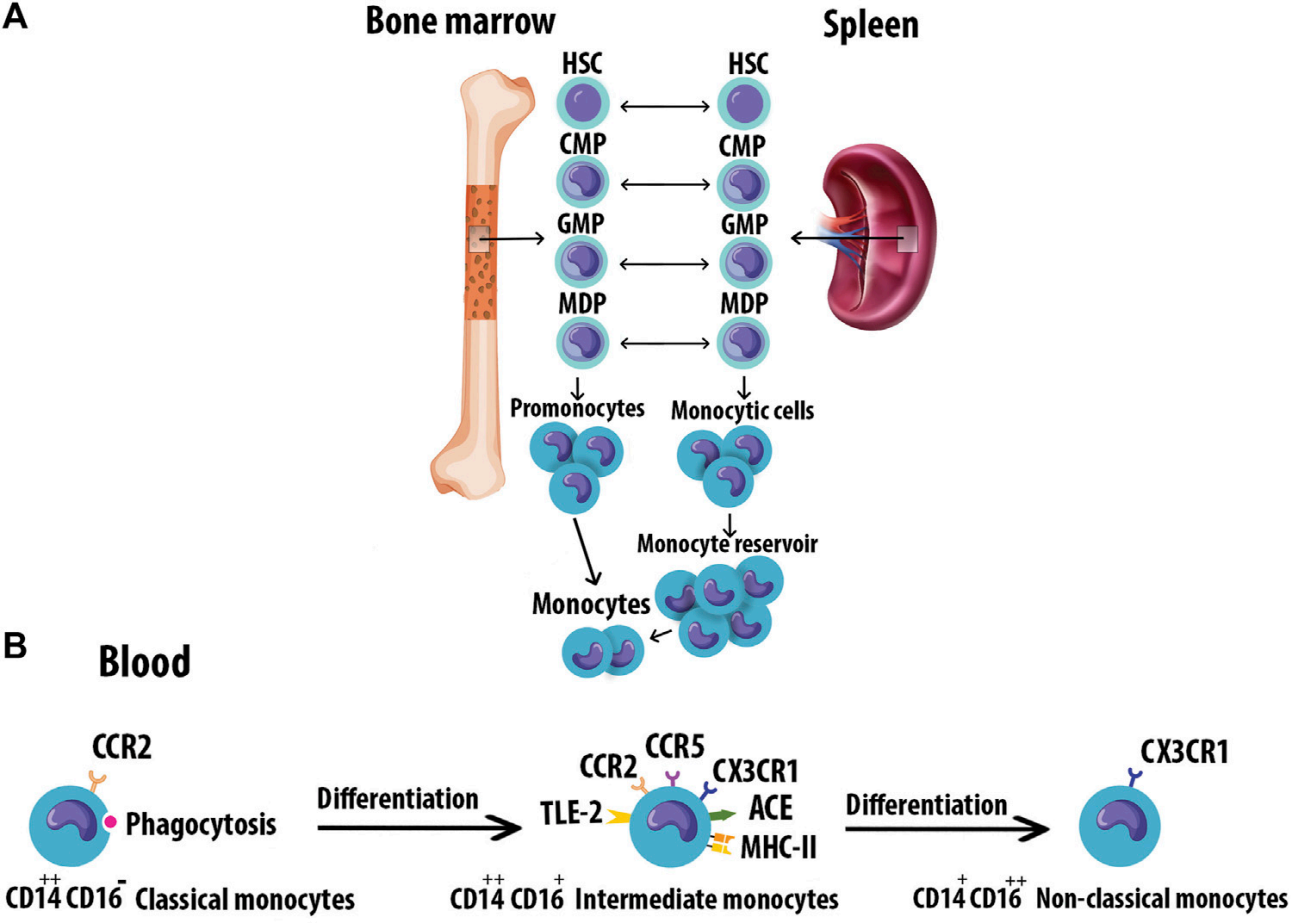
Monocyte origin and heterogeneity. **(A)** Blood monocytes originate from HSC-derived progenitors with myeloid restricted differentiation potential. Successive commitment steps in the bone marrow include CMPs, GMPs, and MDPs. MDPs give rise to monocytes. **(B)** Human monocytes are classified into three subtypes based on the differential expression of CD14 and CD16. Monocytes mature in the bone marrow and are subsequently released into the circulation. Progressively, classical monocytes CD14^++^CD16 give rise to non-classical monocytes CD14^+^CD16^++^ through an intermediate step of CD14^++^CD16^+^ monocytes. Hematopoietic stem cell (HSC), Common myeloid progenitors (CMPs), Granulocyte-Mφ precursors (GMPs), Mφ/Dendritic cell progenitor cells (MDPs).

Monocytes originate from hematopoietic stem cell progenitors in the bone marrow. They have also been found to reside in the spleen as a secondary reservoir. Figure 2A depicts the differentiation of blood monocytes from bone marrow progenitors (Teh et al., 2019). The heterogeneous population of monocytes is classified into classical, intermediate, and non-classical subtypes based on the differential expression of CD14 and CD16 (Figure 2B). The characteristics of monocyte subsets are summarized in Table 1 (Kapellos et al., 2019). Evidence supports a causal role of atherogenic lipoproteins, especially LDL, in the pathogenesis of AS.

**TABLE 1:** Characterization of monocyte subsets.

	Classical	Intermediate	Non-Classical
Surface marker	CCR2, CD62L, CD11b, TLR4, CD36, CD64	CCR2, CX3CR1, HLA-DR, CD74, CD163, CLEC10A, GFRA2, CD86, CCR5	CX3CR1, CX3CR4, HLA-DR, LFA-1
Chemotaxis	CCR2/CCL2	CCR2/CCL2, CX3CR1/CX3CL1	CX3CR1/CX3CL1
Cytokine	IL-1, IL-10	TNF*-*α, IL-10	TNF*-*α, IL-1β
Distribution	∼85%	∼5%	∼10%
Function	Phagocytosis, adhesion, migration, anti-inflammatory responses, anti-microbial responses, scavenger activity	Antigen presentation, regulation of apoptosis, transendothelial migration, pro- and anti-inflammatory responses	Complement and FcR mediated phagocytosis, transendothelial migration, adhesion, healing, pro-inflammatory responses, anti-viral responses (Kapellos et al., 2019)

LFA-1, Lymphocyte function-associated antigen 1.

The subendothelial deposition of LDL is likely a key driver of monocyte-ECs adhesion, an early event of AS by activating the overlying EC (Alderson et al., 1986). Factors such as high-fat diets, smoking, and radiation exposure may increase the risk of long-term OS which manifests itself in excessive reactive oxygen species (ROS) generation and LDL oxidation (Poznyak et al., 2021). In lesions, LDL is oxidized to form Ox-LDL which is pro-inflammatory and appears to contribute to AS initiation and progression (Que et al., 2018). For example, Ox-LDL promotes the expression of vascular cell adhesion molecule-1 (VCAM-1) and intercellular adhesion molecules (ICAM-1) by the ECs which attract the leukocytes into the vessel wall (Milioti et al., 2008). Others have suggested that native or aggregated LDL particles are the key drivers of atherogenesis (Boyle, 2005; Libby, 2021).

Activated lesional ECs and platelets express P- and E-selectin while L-selectin is expressed on leukocytes. The major ligand for selectin is P-selectinglycoprotein ligand 1 (PSGL1) which is expressed on leukocytes and binds to all three selectins. The interaction between selectins and PSGL-1 decelerates fast-flowing leukocytes from the central bloodstream and enables circulating leukocytes to adhere to the activated endothelium. Differential expression or glycosylation of PSGL-1 in leukocytes may result in selective recruitment of monocytes or lymphocytes to atherosclerotic lesions (Huo and Xia, 2009). In addition to inflammatory cells, pro-inflammatory mediators such as cytokines and interleukins are also known to contribute to atherogenesis (Kaperonis et al., 2006).

The adhesion molecules, VCAM1 and ICAM1, are overexpressed on activated ECs (Thayse et al., 2020). Integrins mediate firm leukocyte adhesion on ECs through ligation of monocytes or lymphocytes with VCAM1 or ICAM1 by engaging integrin (Ley et al., 2007). PSGL-1 also interacts with chemokine ligand CCL 21 or CCL19 and enhances chemotactic CD4^+^ T cells to the vulnerable plaques. Activated CD4^+^ T lymphocytes secrete interferon-γ (IFN-γ), tumor necrosis factor-α (TNF-α), pro-inflammatory cytokines enhancing immune responses during atherogenesis (Veerman et al., 2007). IFN-β also increases Mφ accumulation in the plaques and accelerates lesion formation (Goossens et al., 2010). Furthermore, activated platelets, a major component of inflammatory lesions, are phagocytosed by monocytes, inducing secretion of pro-inflammatory chemokines such as CCL5. Thus, the presence of activated platelets on the inflamed endothelium may exacerbate pro-inflammatory Mφ activation (Scull et al., 2010). Human IgG1 against a specific Ox-LDL antigenic epitope promoted the regression of atherosclerotic lesions by inhibiting Mφ recruitment and increasing lipid efflux (Schiopu et al., 2007). Monocyte recruitment is also mediated in part by C-C chemokine receptors (CCR)2, CCR5, and CX3C chemokine receptor 1 (CX3CR1) (Tacke et al., 2007).

### Macrophage Lipid Uptake and Foam Cells

Monocytes migrate into the intima by chemotactic activity and differentiate into Mφs. Mφs can bind to the circulating lipids through several SRs, such as SR-A1, CD36 (SR-B2), and the lectin-type oxidized LDL receptor 1 (LOX1/SR-E1) that mediate uptake of modified LDL (Syväranta et al., 2014).

SR-A1 is mostly on Mφs but also present on VSMCs and ECs experiencing OS. SR-A1 binds to the modified LDL and is involved in the subendothelial translocation of LDL (Apostolov et al., 2009). The c-Jun N-terminal kinase 2 (JNK2)-dependent phosphorylation of SR-A promotes the uptake of lipids in Mφs and thus mediates foam cell formation (Ricci et al., 2004). Deletion of SR-A reduces plaque inflammation and progression toward more advanced necrotic lesions, but it does not abrogate lipid uptake and Mφ foam cell formation in ApoE^−/−^ mice (Manning-Tobin et al., 2009). SR-A1 expression in aortic VSMCs also leads to foam cell formation and enhanced apoptosis in transfected VSMCs (Lehtolainen et al., 2000). SR-A1 triggers endocytosis through two clathrin- and caveolae-dependent routes. Uptake of modified LDL by SR-A primarily goes through clathrin-dependent pathway. SR-A-induced apoptosis needs endocytosis by the caveola route, which activates p38 MAPK and JNK2 signaling (Zhu et al., 2011). NF-κB also regulates the expression of SR-A1 which can be stimulated by pro-inflammatory cytokines (Chistiakov et al., 2017).

CD36 has a high affinity for Ox-LDL, and Ox-LDL/CD36 interactions inhibit cell polarization and Mφ migration (Park et al., 2012). CD36 is upregulated in Mφ-derived foam cells in plaques. Advanced glycation end products (AGEs) are also recognized by CD36 as overexpression of this receptor facilitated AGE uptake in CHO cells (Ohgami et al., 2001). CD36 is also involved in inflammatory processes, including AS, negative regulation of microvascular angiogenesis, lipid metabolism, and clearance of ACs (Febbraio et al., 2001). CD36 showed a conserved role in lipid sensorial recognition. Ox-LDL binds CD36 and triggers TLR4/TLR6 complex to promote pro-inflammatory signaling (Hoebe et al., 2005). Myricetin, a natural flavonoid, contributes to decreased accumulation of cholesterol in Mφ foam cells by inhibition of CD36-mediated Ox-LDL uptake in *Ldlr* ^*−/−*^ mice, resulting in a reduction of atherosclerotic lesions (Meng et al., 2019).

LOX-1 augments the uptake of Ox-LDL by Mφs and ECs. Thus, LOX-1 is implicated in atherogenic deposition of lipids and foam cell formation. The expression of LOX-1 is low under normal physiological conditions, but inflammatory modulators, including Ox-LDL, LPS, mitochondrial ROS, angiotensin II, sheer stress, pro-inflammatory cytokines, AGEs, and conditions such as high blood pressure, dyslipidemia, and diabetes mellitus, upregulate LOX-1 in AS (Kattoor et al., 2019). In addition, Ox-LDL and pro-inflammatory molecules like TNF-α upregulate the LOX-1 expression in VSMCs and facilitate VSMCs transformation to foam cells, a major source of plaque foam cells in human atherogenesis (Kattoor et al., 2019). LOX-1 also contributes to endothelial dysfunction, Mφ differentiation, apoptosis, the proliferation and migration of VSMCs, foam cell formation, platelet activation, as well as plaque instability, and subsequent plaque rupture (Xu et al., 2013).

**FIGURE 3:**
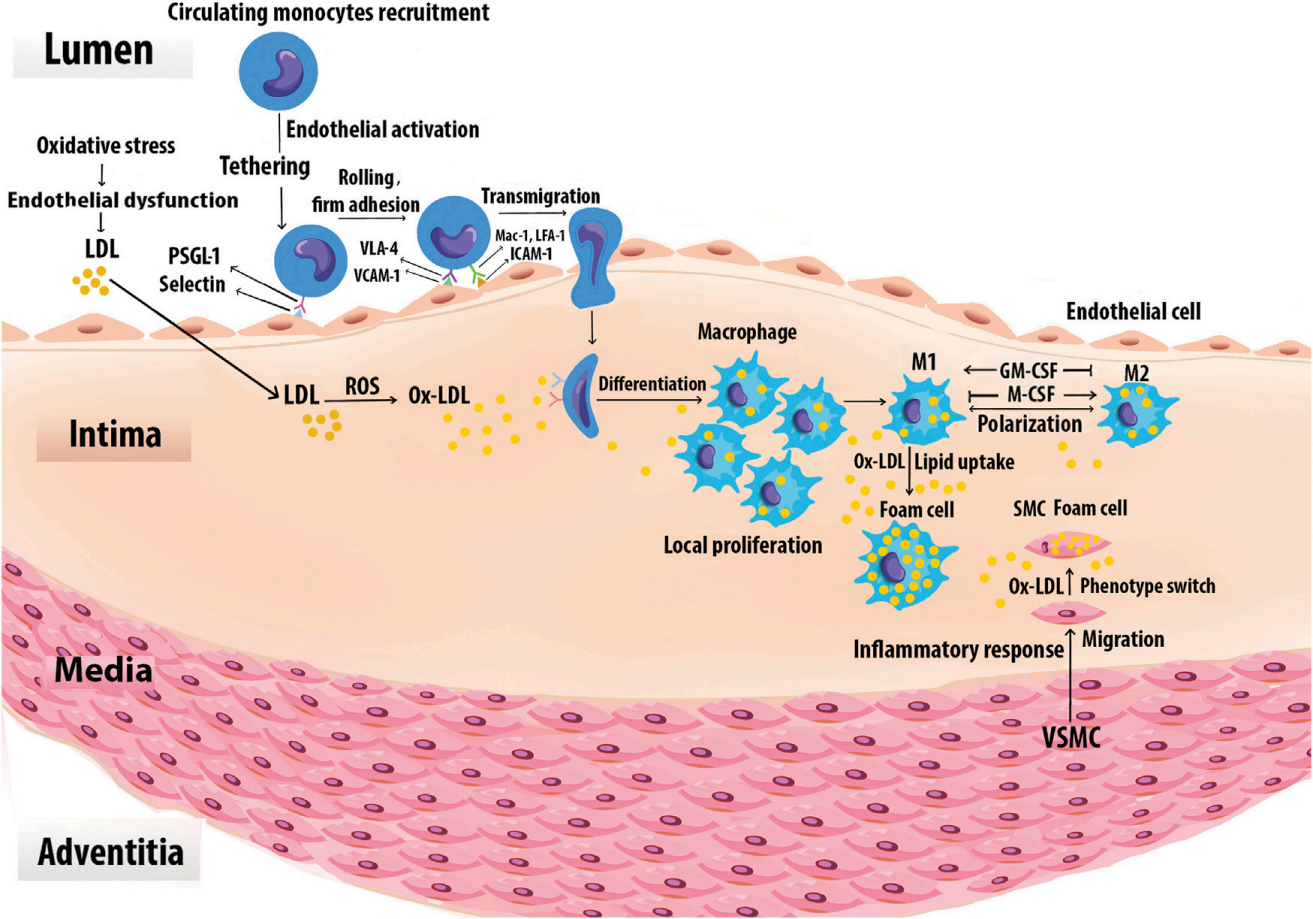
Foam cell formation. Monocytes are recruited to the vascular arginase wall in response to Ox-LDL. Specific adhesion molecules such as the selectins, VCAM-1, and ICAM-1 are expressed on the surface of activated vascular ECs, mediate monocyte adhesion. Once adherent, the monocytes enter the intima and differentiate into Mφs. The differentiation process may be mediated by GM-CSF and M-CSF. Local Mφ proliferation contributes to lesion growth. Upon extensive uptake of Ox-LDL via SRs, Mφs are ultimately turned into foam cells. Chemoattractants, growth factors, and cytokines also promote SMC proliferation, uptake of Ox-LDL, and eventually conversion to foam cells. Foam cells derived from SMCs together with those derived from Mφs generate the fatty lesion.

**FIGURE 4:**
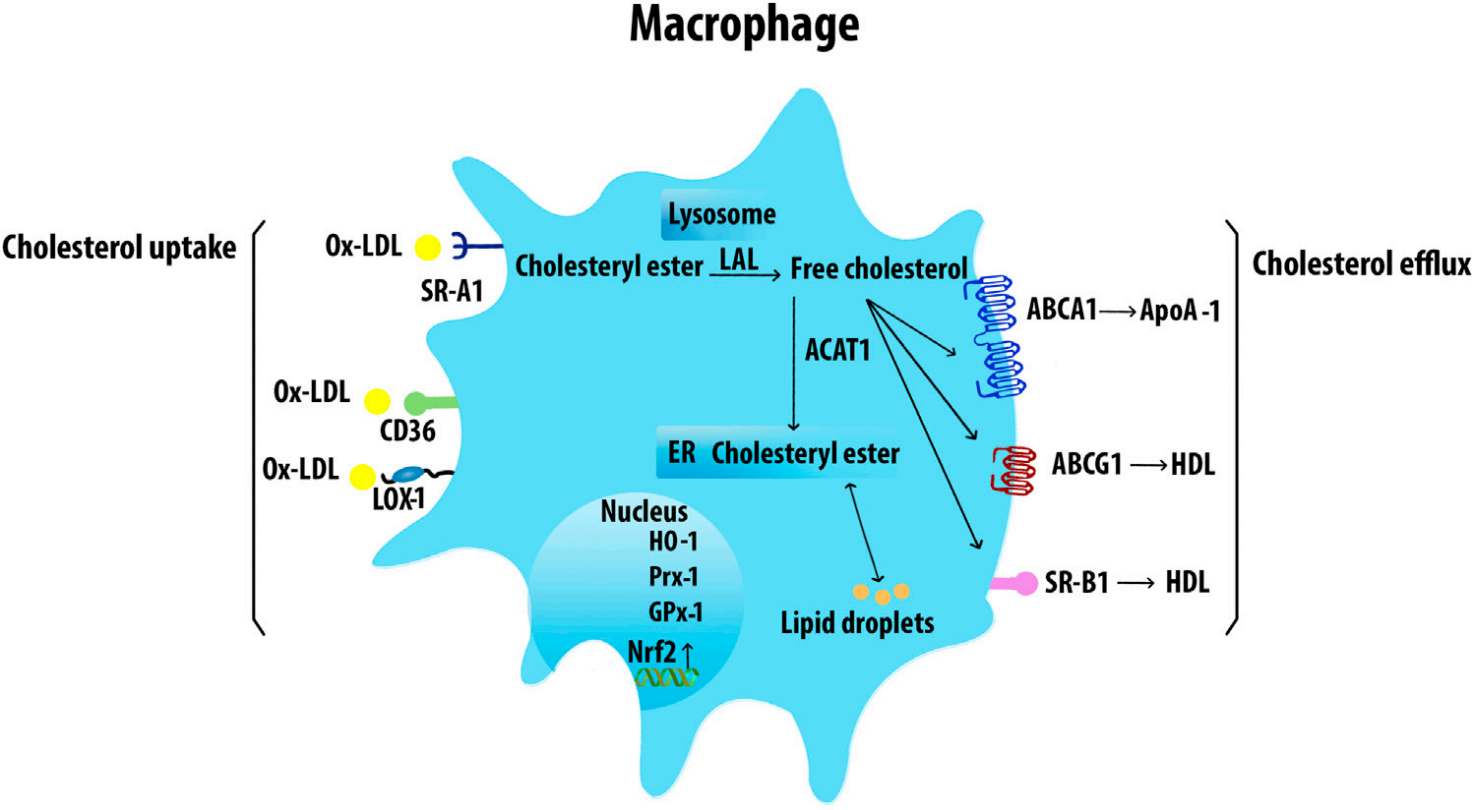
Macrophage cholesterol uptake and efflux. Mφs uptake VLDL and Ox-LDL via SRs including SR-A1, CD36, and LOX-1. The internalized LDL is esterified by acetyl-coenzyme A acetyltransferases and stored in lipid droplets. The ester group is removed from cholesteryl by neutral cholesteryl ester hydrolase through lysosomal acid lipase (LAL) to release FC. ABCA1 transporter mediates the FC efflux from Mφs with ApoA-1. ABCG1 and SR-B1 also efflux FC to mature HDL. Acetyl-CoA acetyltransferase 1 (ACAT1).

The uptake of lipids by Mφs in lesions leads to the formation of cholesterol engorged foam cells (Figure 3). Foam cells are the first sign of initial atherogenesis, although they also play an important role in lesion development and advanced plaques (Chistiakov et al., 2017). The endothelin-1 receptor antagonist directly reduced Mφ lipid accumulation and AS in *Ldlr* ^*−/−*^ mice, indicating the role of endothelin-1 in foam-cell formation (Babaei et al., 2000). The cholesteryl esters in modified lipoproteins are hydrolyzed in lysosomes to free cholesteryl, which is then re-esterified by acetyl-CoA acetyltransferase (Chang et al., 2009) and the accumulating cholesteryl esters are stored as cytoplasmic lipid droplets. Increased cholesterol ester accumulation promotes the development of atherosclerotic lesions, unstable plaque with cap rupture, and thrombosis (Yu et al., 2013). Neutral cholesteryl ester hydrolases are responsible for the removal of the ester group from cholesteryl esters to liberate FC which is then effluxed from cells to HDL through ABCA1, ABCG1, and SR-B1 (Chistiakov et al., 2016). Mφ cholesterol uptake and efflux have been shown in Figure 4.

ABCA1 activity in arterial Mφs plays a major role in the prevention of foam cell formation by mediating the active transfer of cellular phospholipid and FC to apolipoprotein A-1 (ApoA-1), the major apoprotein in HDL (Phillips, 2018). Multiple factors can affect the expression of ABCA1. Extra-virgin olive oil, cineole, quercetin, apelin-13, S-allylcysteine, and TLR2, increase ABCA1 expression, whereas unsaturated FA, miR-26, and IL-12 in synergy with IL-18 inhibit ABCA1 expression.

ABCG1 exports FC or cellular phospholipid to HDL but not to lipid-free ApoA-1. ABCG1 localizes in the plasma membrane, late endosomes, and ER network (Neufeld et al., 2014). Fucosterol, resveratrol, extra-virgin olive oil, and cineole increase ABCG1 expression and significantly reduce cholesterol deposits in the arteries.

SR-B1 promotes FC efflux to mature HDL particles via passive diffusion. The conserved function of SR-B1 across species indicates the importance of its regulation for cholesterol efflux. SR-B1-deficient mice exhibit an increase in accumulation of cholesterol-rich HDL that is accompanied by reduced cholesterol in secreted bile and increased susceptibility to AS (Van Eck et al., 2003). In contradistinction, overexpression of SR-B1 in mice accelerates the metabolism of HDL (Kozarsky et al., 1997). SR-B1 also mediates efferocytosis and reduces atherosclerotic necrosis and inflammation. Deficiency of Mφ SR-BI promoted defective efferocytosis signaling via the Src/PI3K/Rac1 signaling, leading to increased plaque size, necrosis, and inflammation (Tao et al., 2015). Multiple factors were identified to regulate SR-B1 expression. Resveratrol and 13-hydroxy linoleic acid increase the expression of SR-B1, ABCA1, and ABCG1. Caffeic acid and ferulic acid appear to have an antiatherogenic effect by increasing the expression of SR-B1 and ABCG1. Omega-3 fatty acids also induce the activation of SR-B1, ABCA1, and ABCG5, enhancing cholesterol efflux from Mφs (Yu et al., 2013). Risk factors such as infections and fatty diets can change the gut microbiota habitat and increase the levels of circulating LPS which reduces ABCA1/ABCG1 and SR-B1 expression and cholesterol efflux from Mφs (Yu et al., 2013).

### Macrophage Phenotypes in Atherosclerosis

**FIGURE 5:**
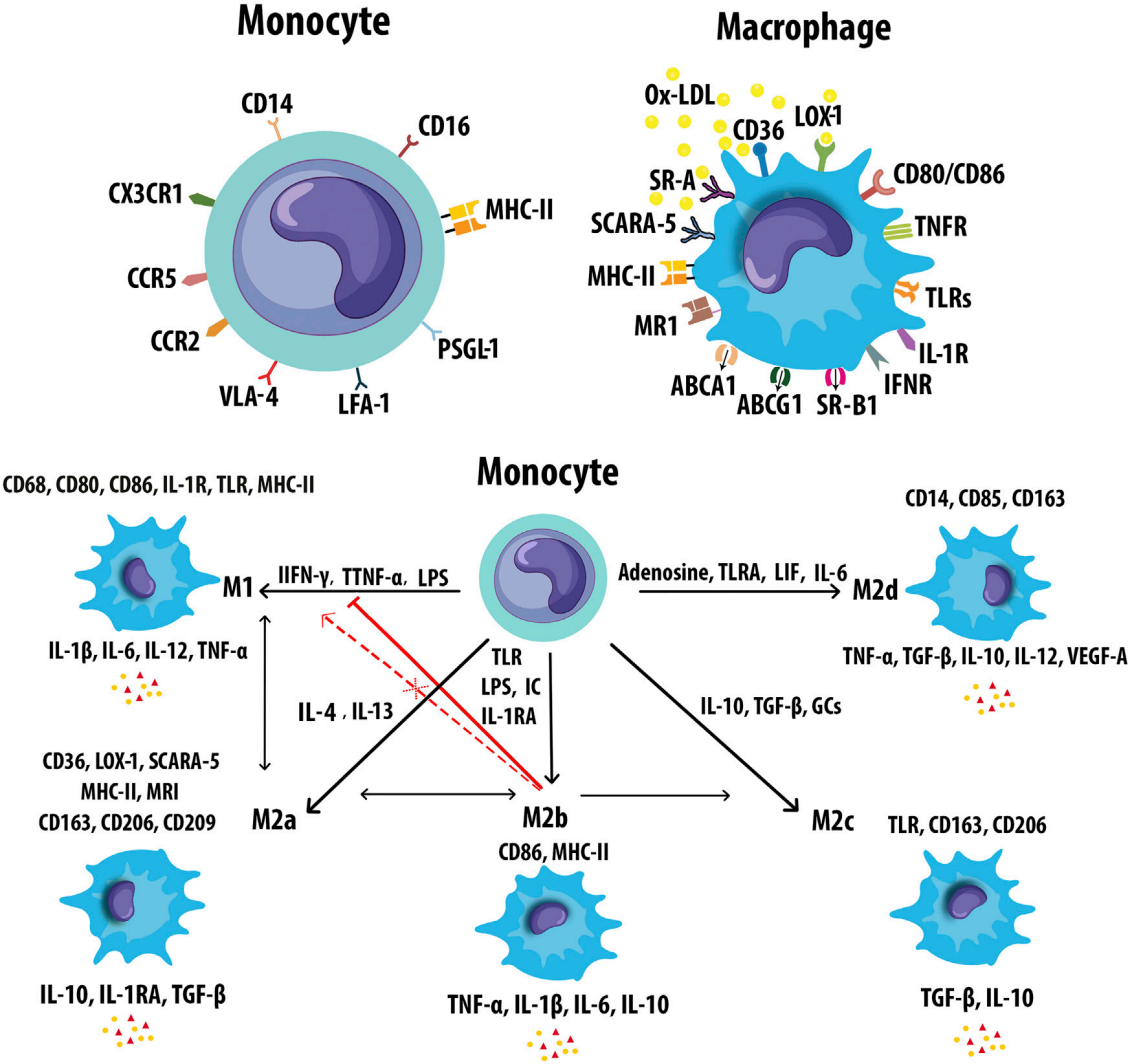
Markers of monocyte and macrophages. **(A)** Monocytes and macrophages express an ample variety of receptors that modulate monocyte and Mφ activation. **(B)** Mφs can polarize to M1 or M2 (M2a, M2b, M2c, and M2d) in response to different inducers and secrete pro- and anti-inflammatory cytokines respectively. M2b Mφs can convert to the other M2 subtypes in response to activated factors. M2b Mφs inhibit the conversion from monocyte to M1 Mφs. M2b also cannot repolarize to M1 during exposure to M1 inducer. Leukemia inhibitory factor (LIF), mannose receptor (MR).

Monocytes/Mφs play a central role in innate immune responses, expressing a variety of receptors that modulate their activation. The expression of monocyte and Mφ markers has been shown in Figure 5A. Mφ polarization refers to a process by which Mφs can differentiate into distinct functional phenotypes in response to microenvironmental stimuli, such as cytokines, chemokines, growth factors, and pathogen-derived molecules (Murray, 2017). M-CSF in early lesions induces anti-inflammatory M2 (M2a, M2b, M2c, and M2d) Mφs, whereas granulocyte M-CSF (GM-CSF) expression upon AS development promotes polarization of pro-inflammatory M1 phenotypes (Colin et al., 2014). Mφ activation states and markers associated with distinct activation phenotypes have been shown in Figure 5B. The dynamic plasticity of Mφs is achieved by transcriptional regulation and thus, specific genes are associated with each type of Mφs (Gerrick et al., 2018). The most abundant T cells in atherosclerotic plaques are Th1 cells and memory CD4^+^ T cells. The Mφ-directed immune response includes both M1/Th1 and M2/Th2 responses. Th1 and Th2 cells in lesions release Mφ-polarizing factors that affect the balancing of M1 and M2 Mφ phenotypes (Bartlett et al., 2019). Both M1 and M2 Mφs contribute to plaque establishment, while M1 and Th1 cells promote plaque destabilization (Bartlett et al., 2019). M2 Mφs were reported to be found at early stages of AS but showed a switch to M1 phenotypes in advanced lesions of *ApoE* ^*−/−*^ mice (Khallou-Laschet et al., 2010). The mixture of Mφ subsets likely exists simultaneously within atherosclerotic aortas *in vivo*, contributing to the progression and persistence of atherosclerotic lesions (Butcher and Galkina, 2012). Gene signatures of Mφ activation are highly robust in predicting inflammation, disease susceptibility, and outcomes, suggesting that immune diversity is a valuable parameter in translational research (Buscher et al., 2017). M1/M2 Mφ subpopulations fail to reflect the full complexity of microenvironments in the plaque. A wide range of cytokines and growth factors are present in AS that could affect the phenotype and polarisation state of Mφs. Atherosclerotic plaques contain other Mφ phenotypes include metabolically activated (MMe) Mφs, oxidized (Mox) Mφs, hemoglobin-related (HA-mac, M(Hb), and Mhem) Mφs, M4 Mφs, lipid metabolism-related trigger receptor expressed on myeloid cells 2 (TREM2, also called lipid-associated Mφs), and neuroimmunological Mφs including nerve-associated Mφs (NAM), and sympathetic neuron-associated Mφs (SAM) (Nagenborg et al., 2017; Kolter et al., 2020b; Porsch et al., 2020). The main properties and functions of polarized Mφ subtypes are summarized in Table 2.

**TABLE 2:** Activating stimuli, properties, and functions of polarized macrophage subtypes.

Subtypes		Inducers	Secreted factors and related genes	Functions
M1		GM-CSF, TNF-α, IFN-γ, endogenous signals (e.g., Ox-LDL, HSP, HMGB1), bacterial stimuli (e.g., LPS, lipoproteins, dsRNA, LTA)	IL-1, IL-6, IL-12, IL-23, TNF, CXCL8, CXCL9, CXL10, CXCL11, CXCL16, CCL2, CCL3, CCL5	Pro-inflammatory responses
M2	M2a	IL-4, IL-13	IL-1R, IL-10, TGF-β, CCL17, CCL18, CCL22, CCL24	Tissue repair
	M2b	LPS, Immune complexes + TLR, IL-RA	IL-1, IL-6, IL-10, TNF, CCL1	Immunoregulation
	M2c	TGFβ, IL-10, Glucocorticoids	TGF-β, IL-10, CCL16, CCL18, CXCL13	Efferocytosis
	M2d	TLR Agonists + Adenosine, IL-6, LIF	IL-6, IL-10, IL-12, TNF-α, TGF-β, CCL5, CXCL10, CXCL16, CCL18	Wound healing, Angiogenesis, Development of tumors (Cheng et al., 2019)
MMe		NOX2, OxPLs, Glucose, Insulin, FFAs	IL-6 (NOX2-dependent) (Li et al., 2020b)	Pro-inflammatory effect, Chronic inflammation, Dead adipocyte clearance
Mox		OxPL	IL-1β, IL-10, VEGF, CX3CR1^-^ (mouse)	Phagocytosis^L^, Anti-inflammatory action, Pro-inflammatory effect, AS development, Chronic inflammation, Anti-oxidant
M(Hb)		Hb/Hp complex	ABCA1, ABCG1, LXR-α, IL-10	Cholesterol efflux^H^, Formation of foam cell, iron content and ROS product^L^, HB clearance, Atheroprotective (Chistiakov et al., 2015)
HA-mac		Hb/Hp complex	IL-10 (Boyle et al., 2009)	HB clearance, Reduction of OS, Atheroprotective
Mhem		Heme	LXR-β, ABCA1, ABCG1 (Medbury et al., 2014)	Erytrophagocytosis, Atheroprotective
M4		CXCL4	TNF-α, IL-6, CCL18, CCL22	Weak phagocytosis, Minimal foam cell formation Fibrous cap degradation, Proatherogenic action (Chistiakov et al., 2015)
TREM2		LDL, ApoE, (Deczkowska et al., 2020), Nucleotides released from damaged cells (Kober and Brett, 2017)	SPP1, RNASE1, MT1G, SEPP1, FOLR2, NUPR1, KLHDC8B, CCL18, MMP12, ApoC2, and complement system genes (C3, C1QA, C1QB, C1QC) (Xiong et al., 2020)	Lipid metabolism, OS, Lesion calcification, Marker of TAMs, Immunosuppressive activity, Regulation of phagocytosis, proliferation, survival (Porsch et al., 2020; Xiong et al., 2020)
SAM		NI	NI	Pro-inflammatory effect, Thermogenesis, Obesity, NE homeostasis
NAM		NI	NI	Weak energy metabolism with age and obesity, NE homeostasis (Kolter et al., 2020a)

HMGB1, High-mobility group box 1tbox1 protein; LTA, lipoteichoic acid; NOX2, NADPH-oxidase-2; VEGF, vascular endothelial growth factor; Hb/Hp complex, Hemoglobin–haptoglobin complex; LXR, Liver X receptor; SPP1, Secreted Phosphoprotein 1; RNASE1, Ribonuclease A Family Member 1; MT1G, Metallothionein-1G; SEPP1, selenoprotein P plasma 1; FOLR2, Folate receptor 2; NUPR1, Nuclear Protein 1; KLHDC8B, Kelch Domain Containing 8B; C3, Complement component 3; C1QA, C1QB, C1QC, Complement C1q A, B, C Chains; TAMs, Tumor-associated Mφs; NE, norepinephrine; H, high; L, low; NI, not identified.

### M1 Macrophages

**FIGURE 6:**
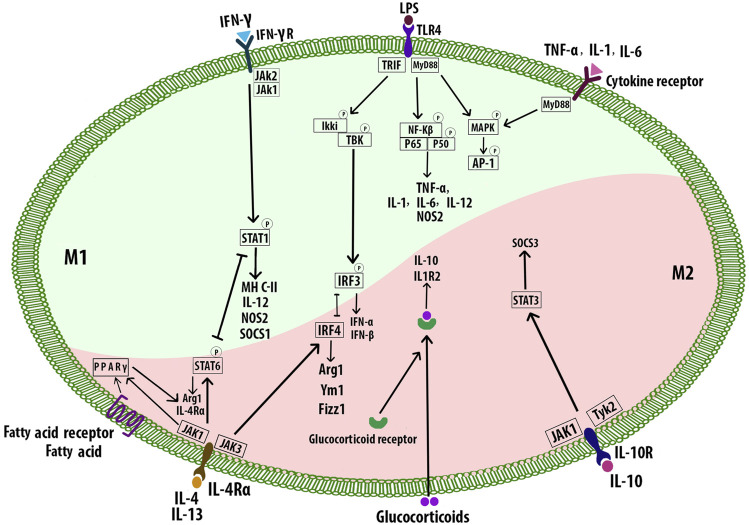
Macrophage polarization signaling pathways. M1 phenotypes express transcription factors such as NF-κB, STAT1, AP-1, and IRF3. M2 subtypes are stimulated by the Th2 cytokines IL-4 and IL-13 which bind to the receptor IL-4Rα and cause the activation of STAT6, IRF4, and PPAR-γ. The intracellular glucocorticoids also promote the transcription of anti-inflammatory genes. IL-10 activates STAT3 and subsequent upregulation of SOCS3. Signal transducer and activator of transcription 1 (STAT1), interferon regulatory factor -3 (IRF3), nitric oxide synthase 2 (NOS2), suppressor of cytokine signaling 3 (SOCS3), arginase 1 (Arg1).

Th1 cytokines including IFN-γ, TNF-α, GM-CSF, and also bacterial stimuli such as LPS polarize Mφs toward the M1 phenotype (Nikonova et al., 2020). M1 cells secrete high levels of pro-inflammatory factors including interleukin-1β (IL-1β), IL-6, IL-12, IL-23, low levels of IL-10, and chemokines such as CXCL-9, CXCL-10, and CXCL-11. M1 Mφ polarization is a tightly controlled process including a set of signaling pathways. Toll-like receptor 4 (TLR4) can be stimulated by LPS or other microbial ligands. This, in turn, promotes activation of interferon regulatory factor-3, activator protein 1 (AP-1), and NF-κB. Myeloid differentiation factor 88 (MyD88), one of the adaptors in responding to TLR4 activation, triggers NF-κB pathway (p65 and p50). This pathway regulates inflammatory genes including pro-inflammatory cytokines such as TNF-α, IL-6, and IL-12. MyD88 activates AP-1 via MAPK signaling. Enhanced AP-1 expression is also mediated by cytokine receptors. Signal transducer and activator of transcription 1 (STAT1) is activated by IFN-γ receptor which regulates the expression of specific genes such as MHC-II, IL-12, nitric oxide synthase 2, and the suppressor of cytokine signaling to promote the M1 Mφ polarization (Figure 6) (Wang et al., 2014b). Mφs express Akt1, Akt2, and Akt3 isoforms which are vital for cell proliferation, migration, and survival (Babaev et al., 2014). Akt1 and Akt2 play opposing roles in Mφ polarization. Akt1 deficiency inducing M1 and Akt2 ablation resulting in M2 phenotype (Arranz et al., 2012). Deficiency of Akt2 suppressed the ability of Mφ to undergo M1 polarization reducing the formation of both early and advanced AS in *Ldlr* ^*−/−*^ mice (Babaev et al., 2014). While deletion of Akt1 resulted in enhanced AS and occlusive coronary artery disease in *ApoE* ^*−/−*^ mice (Fernández-Hernando et al., 2007). Mice with reduced mitochondrial oxidative phosphorylation (OxPhos), showed increased M1-like Mφ polarization and decreased Th2 cytokine responsiveness (Jung et al., 2018). Activated M1 Mφs also produce ROS and nitric oxide (NO) through NADPH oxidase system, which may regulate the phagocytosis process of Mφ, disrupting normal cell metabolism, inducing apoptosis, and ultimately causing chronic tissue damage and plaque formation (Xu et al., 2019). M1 Mφs are enriched in lipids and implicated in initiating and sustaining inflammation, atherosclerotic lesion enlargement, and promoting unstable plaques. M1 Mφs are also the most abundant cells in the lesions of infarction and coronary artery disease (CAD) patients (Barrett, 2020).

### M2 Macrophages

Alternatively, activated M2 Mφs accumulate at the site of injury and release mediators that suppress inflammation in plaques, clear apoptotic cells (ACs), promote allergy, angiogenesis, tissue repair, fibrosis, and plaque stability (Wynn and Vannella, 2016; Barrett, 2020; Miki et al., 2021). M2 phenotypes are stimulated by the Th2 cytokines including IL-4 and IL-13 which bind to the receptor IL-4Rα and promote the M2 polarization through several pathways such as JAK1 and JAK3 signaling which further result in the activation of STAT6, IRF4, and peroxisome proliferator-activated receptor γ (PPAR-γ). STAT6, IRF4, and PPARγ regulate many of the genes such as *Arg1*, *Fizz1*, and *Ym1* associated with mouse M2 Mφs. The fatty acid receptor also activates PPARγ. Inhibition of PPARγ with its antagonist blocked convallatoxin-induced M2 Mφ polarization and enhanced inflammatory responses (Wang et al., 2014a; Zhang et al., 2021). Convallatoxin is a natural cardiac glycoside that protects against AS. Intracellular glucocorticoids bind the glucocorticoid receptor resulting in the nuclear translocation of the complex. The complex binds DNA, promoting the transcription of anti-inflammatory genes such as IL-10 and IL1R2. Alternatively, the complex can also interact with other transcription factors such as NF-κB or AP-1. IL-10 can be produced by all leukocytes, and IL-10 receptor ligation leads to activation of the transcription factor STAT3 that upregulates the expression of SOCS3 which in turn mediates the suppression of pro-inflammatory cytokine signaling (Figure 6) (Wang et al., 2014a). Akt activation is required for M2 activation (Covarrubias et al., 2015). Several signals such as IL-10, and TGF-β promote M2 polarization via PI3K/Akt signaling (Park et al., 2011; Gong et al., 2012). Akt2^−/−^ Mφs attributed to the reduced level of miR-155, which targets C/EBPβ, a key regulator of Arg1 expression and M2 polarization (Arranz et al., 2012). Different stimuli have been associated with the differentiation of M2 subtypes. IL-4, IL-13, and also high levels of CD206 and IL-1 receptor antagonist induce M2a which has anti-inflammatory and tissue remodeling properties. M2b is affected by immune complexes in combination with IL-1β and LPS, and it is involved in immunoregulation. IL-10 and TGFβ or glucocorticoids drive M2c, which has a role in efferocytosis and tissue remodeling. M2b and M2c share regulatory functions, characterized by the secretion of IL-10, and suppression of pro-inflammatory cytokines. IL-6, leukemia inhibitory factor adenosine, and TLR signals induce M2d with angiogenesis, and tumor growth effects (De Paoli et al., 2014; Liberale et al., 2017; Raggi et al., 2017; Abdelaziz et al., 2020). M2 cells also induce the expression of specific chemokines CCL24, CCL17, and CCL22 (M2a), CCL1 (M2b), CCL16, and CCL18 (M2c) (Mantovani et al., 2004). Overall, M2-derived chemokines and cytokines recruit tissue repair cells. Reducing Mφ-inflammation by modulating inflammatory cytokine secretion can promote plaque regression (Lin et al., 2021). M2 Mφs can affect plaque regression via anti-inflammatory effects (Moore et al., 2013) and promote wound healing and tissue repair through collagen formation (Spiller et al., 2014) and removal of dead cells by efferocytosis (Chang et al., 2015). Thus, regulating Mφ polarization to the M2 phenotype could be a therapeutic strategy to prevent the progression of AS or even hasten regression of plaques (Bi et al., 2019). In mouse models of AS regression, the plaque content of M2 markers increased, while M1 markers decreased. Lesions enriched in M2 Mφ are characterized by less inflammation and more stabilizing material, increased content of collagen, and a reduction in the cholesterol content in the plaques (Feig et al., 2011). Administration of IL-13, which is an M2 polarizing factor, protected from AS by increasing lesional collagen content and reducing VCAM-1-dependent monocyte recruitment in *Ldlr* ^*−/−*^ mice (Cardilo-Reis et al., 2012). AS regression after lipid-lowering is dependent on the recruitment of Ly6C^hi^ inflammatory monocytes and their STAT6-dependent polarization to the M2 subtype in mice. The local accumulation of Mφs are highly efficient in clearing ACS. M2 subtype Mφ localized in areas of neovascularization, outside the lipid core, and can phagocytose apoptotic M1 phenotype Mφ, contributing to the resolution of inflammation and inhibition of necrotic core formation within lesions (Chinetti-Gbaguidi et al., 2015).

### Other Macrophage Phenotypes

Mφ differentiation into MMe and Mox phenotypes in adipose tissue (AT) is induced by cytokines and OxPLs derived from Ox-LDL which play important roles in the development of chronic inflammation (Haka et al., 2016). NADPH-oxidase-2 is the main driver of MMe Mφs functions (Coats et al., 2017). The Mox phenotype is associated with the expression of surface markers including *Srnx-1* and *Txnrd-1* and it is abundant in advanced plaques comprising CD68^+^ cells (Kadl et al., 2010; Leitinger and Schulman, 2013).

HA-mac and M(Hb) are atheroprotective phenotypes that are induced by a hemoglobin-haptoglobin complex, implicated in hemoglobin clearance in hemorrhagic sites. M(Hb) Mφs regulate intracellular lipid balance by increasing cholesterol efflux mediators, thereby preventing the formation of foam cells (Chistiakov et al., 2015). Since Mφ intracellular iron levels may drive cholesterol efflux in M(Hb) cells, the manipulation of Mφ iron levels can be useful to retard AS development by upregulation of Mφ cholesterol efflux (Habib and Finn, 2014). Following endocytosis of hemoglobin-haptoglobin complex, heme is released from RBCs which stimulates Mφ polarization to human atheroprotective Mhem subtype implicated in erythrophagocytosis. The Mhem Mφ suppressed OS, lipid accumulation, and foam cell formation, sharing properties with M (Hb) (Skuratovskaia et al., 2020).

M4 Mφs are induced by CXCL-4 in human atherosclerotic plaques. M4 Mφs have pro-inflammatory and proatherogenic properties which may develop in arterial thrombosis (Skuratovskaia et al., 2020). M4 Mφs are unable to efficiently phagocytose Ox-LDL, with minimal formation of foam cells, and also may not function as efferocytes within plaques (Butcher and Galkina, 2012). They can be involved in fibrous cap degradation, and plaque rupture by producing the enzyme MMP12. M4 Mφ also seems to be irreversible and can not switch like M1, M2, or hemorrhage-associated phenotypes (Skuratovskaia et al., 2020).

TREM2 is a main determinant of adipose tissue homeostasis and a modulatory receptor involved in the regulation of phagocytosis, proliferation, and cell survival. TREM2 Mφs correspond to foamy lipid-laden Mφs accumulating in atherosclerotic plaques. Hematopoietic TREM2 is overexpressed on a subset of AS-associated aortic Mφs and is associated with lipid metabolic processes, OS, and lesion calcification while downregulating pro-inflammatory genes (Porsch et al., 2020).

Neuroimmunological Mφs have been reported in different tissues such as the sciatic nerve, adipose tissue, intestine, skin, and microglia which are implicated in thermogenesis, norepinephrine homeostasis, and obesity (Kolter et al., 2020a). Obesity may increase the risk of OS, and subsequent oxidation of LDL particles and atherogenesis (Poznyak et al., 2021). Neuroimmunological Mφs may have therapeutic potential for individuals with obesity (Skuratovskaia et al., 2020).

## Bacterial and Viral Mediators of Macrophage Function

Bacterial and viral mediators are modulators of Mφ lipid metabolism and AS. ECs and Mφs in atherosclerotic plaques upregulate the expression of TLR in response to microbial antigens followed by inflammatory signals leading to AS. There is evidence that TLR3/4 ligands inhibit cholesterol efflux from Mφs leading to increased susceptibility to AS (Castrillo et al., 2003). *Porphyromonas gingivalis* induces its uptake by Mφs and stimulates the formation of foam cells, the hallmark of early atherogenesis (Giacona et al., 2004). Similar mechanisms have been observed for Chlamydophila pneumoniae infection where surface antigens such as LPS and heat shock protein (HSP) participate in this process. LPS has been shown to promote Mφs development into foam cells and chlamydial HSP60 may induce LDL oxidation on the lesion site (Milioti et al., 2008). The role of *H. pylori*, in Mφs differentiation into foam cells in a TLR-2- and TLR-4-dependent way has also been reported (Li B. et al., 2020). During *H. pylori* infection, Mφs are typically polarized to M1 phenotype which induces pro-inflammatory cytokines and promotes AS (Quiding-Järbrink et al., 2010; Vijayvergiya and Vadivelu, 2015).

Transcriptome analysis suggests that human cytomegalovirus reprograms monocyte differentiation toward pro-inflammatory M1 Mφs following infection. The upregulation of monocyte migration is necessary for the hematogenous spread of the virus and as a consequence, could promote AS associated with human cytomegalovirus infection (Smith et al., 2004; Chan et al., 2008). The enterovirus receptor CXADR is upregulated in Mφs in atherosclerotic plaques. CXADR expression was associated with M1 Mφ polarization and foam cells formation, suggesting a mechanism by which enterovirus may infect the cells in atherosclerotic lesions (Nilchian et al., 2020).

## Macrophage-Related Cytokines in Atherosclerosis

### Pro-Inflammatory Cytokines

Mφs release cytokines such as IL-1, IL-6, IL-8, IL-12, IL-18, soluble CD40L (sCD40L), and, TNF in response to various inflammatory stimuli. These factors exhibit a variety of effects, discussed below, and appear to induce inflammation-mediated increases in vascular permeability (Arango Duque and Descoteaux, 2014; Jansen et al., 2016).

IL-1 signaling is proatherogenic and implicated in atherothrombosis. The isoforms IL-1α and IL-1β use a shared IL-1R1. Mice deficient in IL-1β show reduced atherosclerotic disease severity (Arango Duque and Descoteaux, 2014). Canakinumab Anti-inflammatory Thrombosis Outcomes Study (CANTOS) measured the efficacy of IL-1β inhibition in reducing CV event rates (Ridker et al., 2011). Targeting IL-1β by monoclonal antibodies inhibited atherosclerotic plaque formation and the progression of AS (Bhaskar et al., 2011). IL-1β induces angiogenesis by recruitment of myeloid and endothelial lineage cells (Carmi et al., 2009). Deficiency of IL-1α inhibited early lesion formation and inflammation in the ApoE^−/−^ mice (Kamari et al., 2011).

IL-6 is associated with multiple inflammatory disorders. IL-6 can bind to the membrane-bound IL-6R or soluble IL-6R. The CANTOS trial showed that modulation of the IL-6 signaling after taking canakinumab, a human anti-IL-1β monoclonal antibody, is associated with decreased CV events independent of lipid-lowering (Ridker et al., 2018). Senescence-associated IL-6 is upregulated in several tissues and may accelerate atherogenesis due to aging-related alterations. Thus, the blockade of IL-6 might be an effective strategy to reduce AS in old people (Tyrrell and Goldstein, 2020). The MIRACL study showed that the levels of high-sensitivity C-reactive protein (hs-CRP), serum amyloid A protein (SAA), and IL-6 inflammatory markers were related to the risk of stroke (Kinlay et al., 2008). A phase II clinical trial demonstrating ziltivekimab, a fully human monoclonal antibody targeting the IL-6 ligand, markedly reduced multiple biomarkers of systemic inflammation and thrombosis including hsCRP, fibrinogen, SAA, sPLA2, and Lp(a). Thus, the direct inhibition of IL-6 might have the potential to maximize anti-inflammatory atherosclerotic benefits (Ridker et al., 2021). Since IL-6 is a pleiotropic cytokine, depending on the target cell type, it can exhibit both pro- and anti-inflammatory properties. IL-6 contributes to pro-inflammatory responses such as Mφ and neutrophil chemotaxis, induction of chemokine, adhesion molecule production, and promotes vascular endothelial growth factor production and conversely anti-inflammatory functions such as inhibition of IL-1 production, and induction of IL-1R antagonist indicating that IL-6 can act to reduce inflammation and protect the CV system. IL-6 promotes the development of CV disease through activation of ECs, induction of VSMC proliferation, pro-thrombotic effects on platelets, and accumulation of Mφ lipid (Reiss et al., 2017). A high serum level of IL-6 in intermediate CV risk patients referred for coronary angiography is predictive of CAD (Wainstein et al., 2017).

IL-8 is a proatherogenic cytokine produced by Mφs. Upregulation of IL-8 in atherosclerotic plaques promotes recruitment of monocytes by mediating the rolling of monocytes to adhere firmly to the vascular EC (Apostolopoulos et al., 1996; Gerszten et al., 1999). In addition, elevated serum levels of IL-8 are increased in patients with unstable angina (Romuk et al., 2002). High serum levels of IL-8 are related to an increased risk of all-cause mortality independent of the underlying cause (Velásquez et al., 2019).

IL-12 is formed mainly by plaque Mφs and stimulates the differentiation of CD4^+^ T cells into Th1 cells through the IL-12Rβ2 chain. Th1 cells can further activate Mφs and subsequently the inflammatory cascade (Kleemann et al., 2008). The administration of IL-12 resulted in enhanced lesion size in ApoE^−/−^ mice (Davenport and Tipping, 2003). ApoE^−/−^ mice lacking TLR4 or MyD88 showed a decrease in AS that was associated with a significant reduction in the circulating levels of IL-12 (Michelsen et al., 2004). Exposure of non-stimulated CD4^+^CD28^−^ T cells to IL-12 induced recruitment of T cells into the atherosclerotic plaque in human atheroma-SCID mouse chimeras (Zhang et al., 2006). These results suggest that IL-12 is a proatherogenic and pro-inflammatory cytokine.

IL-18 acts as a pro-inflammatory cytokine by mediating the production of IL-1β, IL-8, and the expression of ICAM-1 and VCAM-1 (Dinarello et al., 2013). IL-18 accelerated AS by upregulation of CD36 and MMP-9 expression via NF-κB pathway in ApoE^−/−^ mice. NF-κB blockade inhibited IL-18 signaling through downregulation of IL-18, IL-18Rα, CD36, and MMP-9, and upregulation of LXR-α resulted in protection against IL-18-induced AS (Bhat et al., 2015). The proatherogenic effect of IL-18 in the absence of T cells is accompanied by elevation of IFN-γ and CXCL16 expression (Tenger et al., 2005). High serum concentrations of IL-18 increase the risk of future coronary heart disease (Jefferis et al., 2011).

CD40 is expressed mostly on the M1 phenotype (Jansen et al., 2016). Plasma CD40 levels are associated with the severity of carotid AS and an increased risk for future CV events. Intra-plaque levels of sCD40 and sCD40L are also associated with vulnerability and remodeling (Shami et al., 2020). The co-stimulatory CD40 and CD40L are implicated in the regulation of the inflammatory response during AS development. Targeting non-classical CD40L-Mac1 interactions and the inflammatory CD40-TRAF6 signaling may have the potential to reduce the residual inflammatory risk that drives AS (Bosmans et al., 2020).

TNF activates ECs to induce the expression of multiple adhesion molecules and promotes secretion of a variety of inflammatory cytokines and chemokines to enhance the recruitment of activated leukocytes into the lesions (McKellar et al., 2009). TNF-α is a pleiotropic cytokine and functions through its two main receptors including TNF receptors 1 and 2 (TNFR1 and TNFR2). Pro-inflammatory signaling, apoptosis, and degenerative cascades are reported to mediate through TNFR1 while TNF-α signaling through TNFR2 activation is anti-inflammatory and cytoprotective, leading to cell proliferation, differentiation, angiogenesis, and tissue repair (Subedi et al., 2020). TNF-α is implicated in vascular dysfunction. The increase of TNF-α expression by AGEs, their receptors, LOX-1, and NF-κB signaling may induce ROS production leading to endothelial dysfunction in CV disease (Zhang et al., 2009). TNF-α enhances the progression of lesions towards an advanced phenotype by stimulating necrosis and decreasing the incidence of apoptosis in transgenic mice (Boesten et al., 2005). Anti-TNF-α therapy and drugs like thalidomide that inhibit TNF-α production may reduce CV events and inhibit the early development of AS (Chew et al., 2003).

### Anti-Inflammatory Cytokines

IL-10 exhibits anti-inflammatory properties that may be protective against AS (Mallat et al., 1999). IL-10 appears to impair HIV-related CD4^+^ T cells and might be a potential target for the treatment of AS in HIV (Fourman et al., 2020). An inverse correlation between AS severity and IL-10^+^ B cells was found in Ldlr^−/−^ mice, while increased cholesterol may mask the protective effects of IL-10^+^ B cells (Douna et al., 2019). AS was attenuated through increased expression of IL-10 in programmed cell death protein 4 (PDCD4)-deficient mice. Thus, PDCD4 could affect AS development by regulating the expression of IL-10 (Jiang et al., 2016).

TGF-β is a pleiotropic cytokine that can be both atheroprotective and atherogenic. Deficiency of growth differentiation factor 15 (GDF15), a member of TGF-β family, attenuated lesion formation in Ldlr^−/−^ mice in a TGFβRII-dependent manner that reduced Mφ chemotaxis (de Jager et al., 2011). Suppression of endothelial TGF-β signaling reduced inflammation and vascular permeability in hyperlipidemic mice and attenuated disease progression (Chen et al., 2019). TGF-β can also elicit atherogenic effects through its actions on VSMCs in early plaque lesions (Low et al., 2019). An inverse relationship between serum TGF-β1 levels and advanced AS has been observed (Grainger et al., 1995). Inhibition of TGF-β signaling promoted the development of atherosclerotic lesions with unstable plaque phenotype, increased inflammatory cells, and decreased collagen content in ApoE^−/−^ mice (Mallat et al., 2001). TGF-β1 increased cholesterol efflux and attenuated Mφ foam cell formation in ApoE^−/−^ mice (Panousis et al., 2001). Deletion of Tgfb1 resulted in reduced VSMC differentiation, accelerated lesion formation, and increased inflammation in heterozygous mice, indicating that TGF-β can protect against AS (Low et al., 2019).

### Programmed Cell Death of Macrophages and Interactions of Efferocytosis Signaling

**FIGURE 7:**
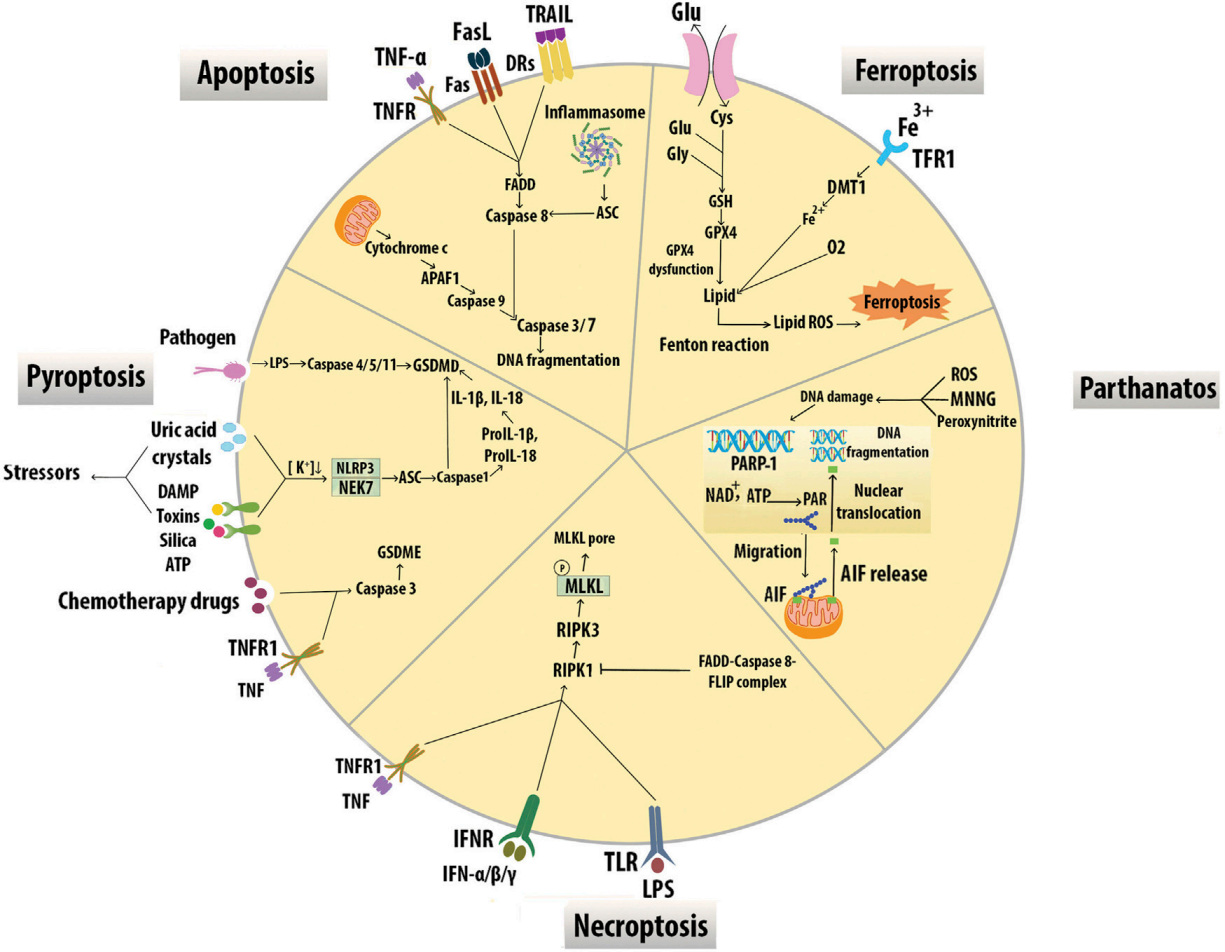
Modes of macrophage death. Different modes of cell death including apoptosis, pyroptosis, necroptosis, parthanatos, and ferroptosis have unique activating stimuli and present different signaling pathways and distinct physiological outcomes. Inhibitors of the divalent metal transporter 1 (DMT1).

Mφ apoptosis, impaired Mφ efferocytosis, and secondary Mφ necrosis have been implicated in necrotic core and vulnerable plaque formation (Gonzalez and Trigatti, 2017). While the most common form of Mφ death is apoptosis, other programmed cell death modalities in Mφ include immune-reactive cell death (pyroptosis), necroptosis, mitochondrial-dependent cell death (parthanatos), and iron-dependent cell death (ferroptosis) (Yan et al., 2020) (Figure 7).

Apoptosis can be triggered by the activation of a mitochondrial pathway by cellular stress or through the activation of death receptors (DRs) at the cell surface. The mitochondrial intermembrane protein, cytochrome c, is released into the cytosol and triggers apoptotic protease-activating factor 1 (APAF1), which in turn activates the serine protease caspase 9. Active caspase 9 stimulates the executioner caspases caspase 3 and caspase 7, leading to DNA fragmentation. Death receptors include TNFR1, FAS receptor, and TNF-related apoptosis-inducing ligand (TRAIL). Receptor ligation promotes the recruitment of adaptor proteins, including FAS-associated death domain protein (FADD), which bind and activate caspase 8. Caspase 8 activates the executioner caspases results in cell death. Inflammasomes can also trigger caspase 8 via apoptosis-associated protein (ASC) leading to DNA damage-induced apoptosis (Boada-Romero et al., 2020). Mφ SR-A has a protective effect in early lesions but proatherosclerotic roles in advanced plaques for induction of Mφ apoptosis and efferocytosis. (Devries-Seimon et al., 2005). STAT1 appears to be an essential component of ER stress/SRA-induced Mφ apoptosis in advanced atheromata (Lim et al., 2008). Overexpression of IL-10 by T cells was reported to suppress STAT1 activity and protect from excessive cell death and plaque necrosis (Pinderski et al., 2002). Transcription factor *Zhx2* deficiency promoted Mφ apoptosis and exhibited a reduction in lesion size and resistance to AS in mice. Evidence including BM transplantation studies also supports this effect of *Zhx2* which is mediated in part by monocytes/Mφs (Erbilgin et al., 2018). A prolonged increase of CHOP levels induces apoptosis through cytoplasmic Ca^2+^ metabolism and suppression of prosurvival molecules like Bcl-2 (Tabas, 2010). In early lesions, efferocytosis prevents cellular necrosis and induces anti-inflammatory pathways through TGF-β and the activation of NF-*κ*B signaling to promote cell survival (Henson et al., 2001). In contrast, ER stress in foam cells induces inflammatory pathways within the lesion by suppressing NF-*κ*B signaling and activating JNK, activator protein-1, ROS, and spliced X-box binding protein 1, which promote apoptosis (Xu et al., 2019).

Pyroptosis results in the death of Mφs in response to bacterial infection, accompanied by activation of inflammasomes and maturation of pro-inflammatory cytokines IL-1β and IL-18. Gasdermin D (GSDMD) pores are the effectors of pyroptosis. Cytosolic LPS binds caspase 4/5/11 to trigger their cleavage of GSDMD and subsequent plasma membrane permeabilization leading to pyroptosis. Inflammasome sensor proteins, such as NLRP3, recognize cellular stressors, including those from bacteria, viruses, toxins, ATP, uric acid crystals, silica, and damage-associated molecular pattern (DAMPs). These stressors activate NLRP3 indirectly through potassium efflux, which leads to NEK7 binding NLRP3 to trigger its oligomerization. NLRP3 subsequently activates caspase-1 via the adaptor protein ASC and cleaves GSDMD resulting in pyroptotic cell death. Active caspase 1 also proteolytically processes IL-1β and IL-18 into their active forms, which are subsequently released from pyroptotic cells (Frank and Vince, 2019). Chemotherapy drugs induce pyroptosis through caspase 3 cleavage of Gasdermin E (GSDME) (Wang et al., 2017). Cleavage of GSDME by caspase 3 may also switch TNF-induced apoptosis to pyroptosis (Rogers et al., 2017). AS risk factors could activate NLRP3 inflammasomes in ECs and Mφs. NLRP3 inflammasome-mediated pyroptosis in the atherosclerotic plaques is correlated with plaque rupture and vascular inflammation, suggesting that NLRP3 inflammasome and related pyroptosis play an important role in the pathogenesis of AS (Zeng et al., 2019).

Necroptosis is activated by death receptors including TNFR1, IFNR, and TLR3/4. Necroptosis is initiated through the activation of RIPK1, which binds and activates RIPK3. RIPK3-mediated phosphorylation of the mixed-lineage kinase domain-like protein (MLKL) results in membrane lysis. This process is inhibited by the activation of caspase 8 and its apoptotic inhibitor FLIP which cleaves RIPK1 to prevent necroptosis (Boada-Romero et al., 2020). The expression of necroptosis mediators RIPK3 and MLKL is upregulated in atherosclerotic plaques, especially in vulnerable plaques supporting the role of RIPK3-mediated Mφ necroptosis in the development of AS (Karunakaran et al., 2016; Tian et al., 2016).

Parthanatos in plaque Mφs is triggered by DNA damaging stimuli such as N-methyl-N′-nitro-N-nitrosoguanidine (MNNG), peroxynitrite, and ROS-dependent activation of poly ADP-ribose polymerase (PARP)1. The PAR polymer is synthesized by PARP1 in response to DNA breaks, migrates to the mitochondria, and releases apoptosis-inducing factor (AIF) which interacts with Mφ migration inhibitory factor and degrades DNA (Robinson et al., 2019). PARP1 inhibition may affect endothelial function, lipid metabolism, foam cell formation, switch from necrosis to apoptosis, and thus play a major role in atherogenesis (Xu et al., 2014).

Ferroptosis is associated with iron-dependent accumulation of lipid hydroperoxides during cell death. Cysteine (Cys_2_) can enter the cytoplasm via xCT and synthesizes glutathione (GSH) with glutamate (Glu), and glycine (Gly). Glutathione peroxidase 4 (GPX4) is an antioxidant enzyme that removes oxidative modifications from lipids. When GPX4 dysfunction, lipid transforms into lipid ROS with O2 and Fe^2+^ via the Fenton reaction (Zuo et al., 2020). Lipid peroxidation, intraplaque hemorrhages, and iron deposition are hallmarks of advanced human plaques, which is indirect evidence for the initiation of Mφ ferroptosis and plaque destabilization. Overexpression of GPX4 decreased lipid peroxidation and inhibited plaque development in ApoE^−/−^ mice indicating the possible role of ferroptosis in CV disease (Guo et al., 2008).

**FIGURE 8:**
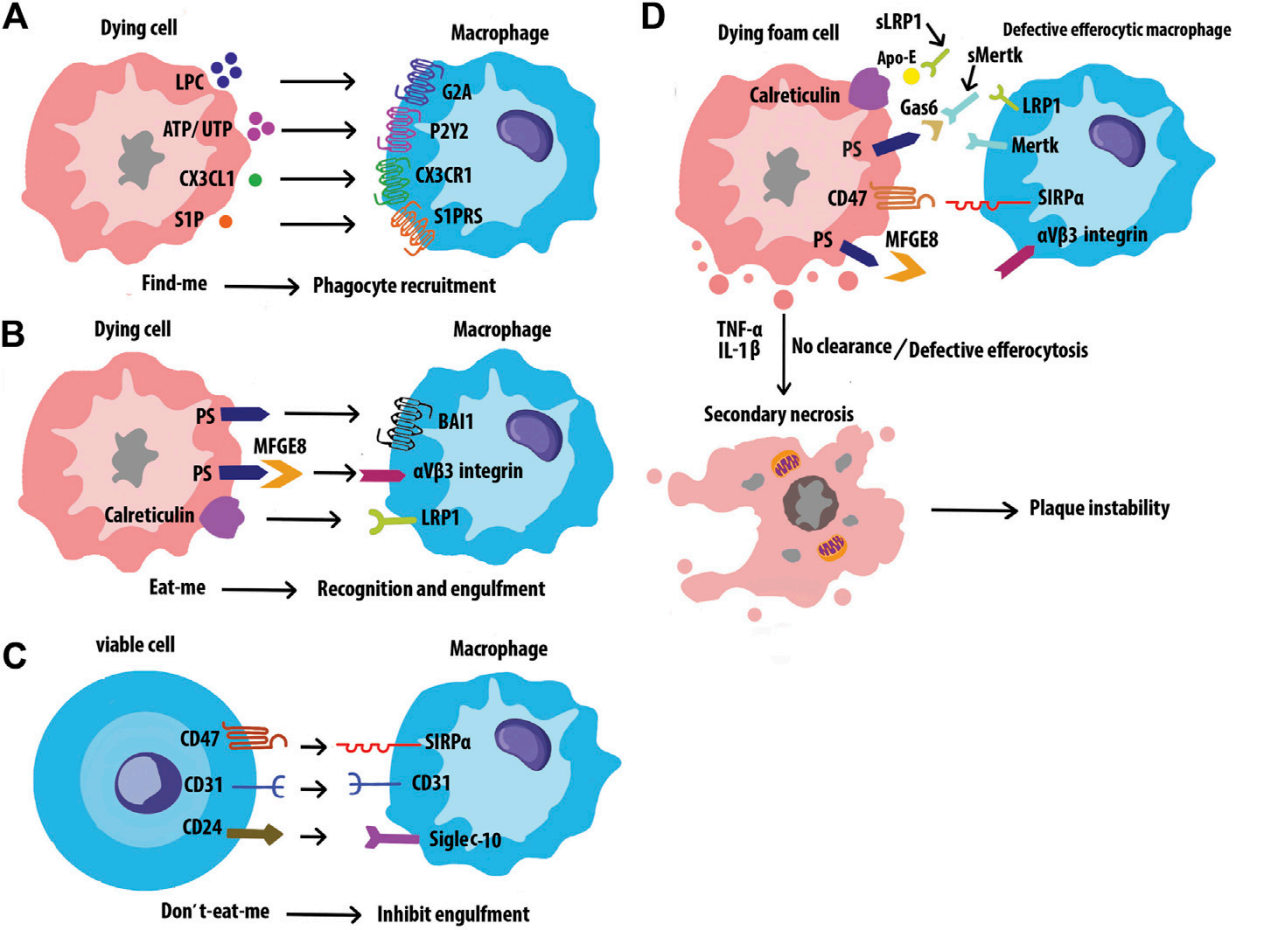
Interactions of efferocytosis signaling. Find-me signals recruit phagocytes to the sites of cell death. The dying cells will express Eat-me signals to facilitate interactions with phagocytes. Non-ACs send a Don’t-eat-me signal to avoid efferocytosis when exposed to phagocytes. Defective efferocytosis leading to inefficient clearance of ACs and subsequent necrosis and inflammation in plaques.

Efferocytosis is the process by which cells undergoing apoptosis are cleared by Mφs, thereby reducing inflammation and maintaining tissue homeostasis (Yan et al., 2020). Disposal of the dying cells requires a variety of signal molecules through which phagocytes recognize and engulf ACs. Find-me signals, such as lysophosphatidylcholine (LPC), ATP/UTP, CX3CL1, and sphingosine-1-phosphate (S1P) are released by ACs, which promote attracting phagocytes to the sites of death. Phagocytes sense these signals via receptors including G2A, P2Y2, CX3CLR, and S1PRs respectively. (Figure 8A). Bridging molecules such as apoE and MFGE8 connect phagocytes to ACs. Afterward, ACs exposed to a variety of signals on their surfaces, interacting with receptors on the phagocytic membrane through Eat-me signals, and efficiently process the AC constituents to maintain homeostasis. The most common Eat-me signal, phosphatidylserine (PS), can interact with a variety of receptors on the surface of phagocytes, such as BAI1 and αvβ3 integrin. Other Eat-me signals, such as calreticulin and ICAM3 modulate the identification and engulfment of ACS via the receptors LRP1 and CD14 respectively (Figure 8B). Healthy cells display don’t-eat-me signals such as CD47, CD31, and CD24, on their surface which binds to receptors SIRP a, CD31, and Siglec-10, respectively expressed on phagocytes to avoid efferocytosis (Figure 8C). Programmed cell removal can be countermanded by anti-phagocytic don’t-eat-me signals such as cell surface expression of CD47. Mouse model studies showed that administration of CD47-blocking antibodies increased intraplaque efferocytosis efficiency and inhibited AS (Kojima et al., 2016). Defective phagocytosis of apoptotic foam cells has several consequences that promote the progression of chronic and non-resolving inflammatory diseases such as advanced AS (Figure 8D) (Wang et al., 2020). Angiotensin Ⅱ is a multifunctional hormone that plays a major role in the development of AS. Angiotensin Ⅱ promotes MerTK shedding via AT_1_R/ROS/p38 MAPK/ADAM17 pathway in Mφs, which leads to defective efferocytosis, accumulation of ACs, and progression of AS (Zhang et al., 2019). Mφs in atherosclerotic plaques express angiotensin Ⅱ type 1 receptor. This receptor of bone marrow-derived Mφs worsens the renal injury-induced AS by shifting the Mφ phenotype to M1 and decreasing the M2 phenotype which leads to impaired efferocytosis and enhanced necrosis (Yamamoto et al., 2011) Efferocytosis signaling molecules have been shown in Table 3.

**TABLE 3:** Efferocytosis signaling molecules.

Role	Molecule	Expression	Receptor
Find-me signals	CX3CL1	Dying cell	CX3CR1
	LPC	Dying cell	G2A
	S1P	Dying cell	S1PRs
	ATP/UTP	Dying cell	P2Y2
Eat-me signals	PS	Dying cell	BAI1/MFGE8-aVβ3 integrin
	Calreticulin	Dying cell	LRP1
	ICAM3	Dying cell	CD14
Bridging molecules	MFGE8	Dying cell	avβ5 integrin/avβ3 integrin
	C1q	Macrophage	SCARF1/aMβ2 integrin
	GAS6	Dying cell/Macrophage	AXL/Mertk
	TSP-1	Dying cell	CD36/avβ3 integrin
Don’t-eat-me signals	CD47	Viable cell	SIRPα
	CD31	Viable cell	CD31
	CD24	Viable cell	Siglec-10 (Wang et al., 2020)

CX3CL1, CX3C chemokine ligand 1; LPC, lysophosphatidylcholine; S1P, Sphingosine-1-phosphate; ATP/UTP, Adenosine Triphosphate/Uridine Triphosphate; CX3CR1, CX3C chemokine receptor 1; G2A, G-protein-coupled receptor; S1PRs, Sphingosine-1 phosphate receptors; P2Y2, Purinergic receptor P2Y2; PS, phosphatidylserine; BAI1, Brain-specific angiogenesis inhibitor 1; MFGE8, Milk fat globule-EGF, factor 8; LRP1, Low-density lipoprotein receptor-related protein 1; C1q, Complement component 1q; GAS6, Growth arrest-specific gene 6 product; TSP-1, Thrombospondin-1; SCARF1, Scavenger receptor F1; AXL, anexelekto receptor tyrosine kinase; Mertk, Mer tyrosine kinase; SIRPa, Signal regulatory protein a; Siglec-10, Sialic acid binding ig like lectin 10.

## Macrophages in Advanced Atherosclerosis

Atherosclerotic lesions typically consist of a lipid core which is covered by a fibrous cap. Matrix composition, thickness, cellularity, the collagen content of fibrous caps, and chronic inflammatory infiltrate are important factors in plaque stability. Vulnerable plaques are associated with active inflammation and a thin fibrous cap (Sodhi and Brown, 2018). Mφs secrete proteolytic enzymes, especially matrix metalloproteinase (MMP)-2 and MMP-9, causing degradation of ECM proteins such as collagen and elastin leading, to fibrous cap thinning and destabilization of advanced plaques. Thus, MMP-9 inhibitors have the potential to stabilize vulnerable plaques (Gough et al., 2006). Lesional Mφs induced apoptosis of VSMCs by TNF-α, the Fas apoptotic pathway, and NO production and appear to promote rupture-prone plaques (Boyle et al., 2003).

A key feature of unstable plaques is the formation of necrotic cores. If efferocytosis is insufficient, dead M1 Mφs accumulate and undergo postapoptotic necrosis, leading to the formation of a necrotic core. The thin fibrous cap and large necrotic core make lesions susceptible to rupture, leading to thrombosis, heart attack, or stroke (Virmani et al., 2002; Chinetti-Gbaguidi et al., 2015).

## Clonal Hematopoiesis and Leukocytosis in Atherosclerosis

Normal circulating blood cells consist of a polyclonal mixture descended from thousands of hematopoietic stem cells (HSC). During aging, or in blood cell cancers, individual HSC clones can become relatively abundant. In 2017–2018, several groups reported that clonal hematopoiesis (CH), defined as expanded somatic blood cell clones in the absence of hematologic abnormalities, dramatically accelerates AS and increases the risk of MI (Fuster et al., 2017; Jaiswal et al., 2017; Sano et al., 2018). These clones were associated with various driver gene mutations such as the epigenetic modifiers TET2 and DNMT3A, suggesting that the mutations not only enhanced cell proliferation but also conferred pro-inflammatory or other properties to the leukocytes. Several subsequent studies on CH and AS have now been reported and they generally support the pro-inflammatory effects of certain driver mutations (Bick et al., 2020; Fidler et al., 2021).

The possibility that these observations with clonal hematopoiesis are due in part to increased hematopoiesis rather than pro-inflammatory clones was raised by Heyde et al. (Heyde et al., 2021). It has long been known that elevated levels of blood leukocytes, a phenomenon termed leukocytosis, predicts CV events, and certain studies have suggested that the presence of a vicious cycle in which events such as MI and AS risk factors promote leukocytosis (Swirski and Nahrendorf, 2013a). Previous studies had shown that the hematopoietic system is activated in AS in a variety of species including mice and humans. The authors showed, using fundamental evolutional dynamics, that this would predict the expansion of clones harboring both advantageous (in terms of increased proliferation) and neutral mutations. The authors quantitatively examined HSC proliferation in atherosclerotic mouse models as well as human subjects using incorporation of bromodeoxyuridine into DNA or the expression of the proliferation marker Ki67. In both atherosclerotic mice and patients with AS, HSC proliferation was markedly increased, roughly 2-fold, as compared to controls. It is noteworthy that smoking, a strong risk factor for AS, promotes CH and leukocytosis. A particularly exciting connection with HSC proliferation and clonal hematopoiesis in AS relates to the role of chronic stress. Sleep modulates hematopoiesis and protects against AS. Previously, McAlpine et al. (McAlpine et al., 2019) showed that sleep fragmentation in mice suppressed hypothalamic secretion of the wake-promoting peptide hypocretin, resulting in elevated M-CSF expression by pre-neutrophils in bone marrow that, in turn, increased blood monocytes and AS. The authors confirm in the present study that sleep fragmentation increases HSC proliferation. Thus, the relative importance in AS of driver mutations such as TET2, as compared to increased hematopoiesis and leukocytosis, is uncertain. Since both are potential therapeutic targets, the answer is of considerable interest.

## Single-Cell Techniques and Macrophage Heterogeneity in Atherosclerosis

Single-cell sequencing technologies can be applied to sequence DNA, RNA, open chromatin, or methylated DNA in single-cells in the context of normal or diseased tissues (Williams et al., 2020). The single-cell sequencing studies reported thus far have relied primarily on RNA sequencing (scRNAseq). In some cases, the studies have been complemented by time of flight mass cytometry analyses. Over the past 3 years, several groups have used scRNAseq to examine leukocyte heterogeneity in various mouse models of AS (Cochain et al., 2018; Kim et al., 2018; Winkels et al., 2018; Lin et al., 2019; Zernecke et al., 2020) and in human carotid AS (Fernandez et al., 2019) and these studies have been recently reviewed (Willemsen and de Winther, 2020; Fernandez and Giannarelli, 2021). ScRNAseq analyses of mouse atherosclerotic lesions identified three main clusters of the Mφs in AS, resident-like, inflammatory, and TREM2hi (Willemsen and de Winther, 2020). The resident-like Mφs, some of which are found in the adventitia, can proliferate and resemble an M2-like phenotype. The inflammatory Mφs are the major component of the Mφ population within the intima of the plaque. They appear to be the main cellular drivers of lesional inflammation and are enriched in both M1-associated genes and Mox-associated transcription factor NRF2. They are mainly non-foamy (Kim et al., 2018). The third subtype, TREM2hi Mφs, are foamy lipid-laden Mφs enriched for lipid metabolic processes, regulation of cholesterol efflux, OS and cellular catabolic processes and. have an M2-like phenotype. In a comprehensive meta-analysis of mouse studies, Ley’s group (Zernecke et al., 2020) confirmed these known Mφ subsets and identified a new Mφ subset resembling peritoneal cavity Mφs. Thus far only one study has examined human lesion (Fernandez et al., 2019). In that study, carotid plaques of clinically symptomatic disease (stroke) were compared to asymptomatic disease and scRNAseq was complemented with mass cytometry. Mφs from the plaques exhibited alternatively activated phenotypes, some of which were associated with plaque vulnerability.

## Macrophage Senescence in Atherosclerosis

Accumulating evidence from both human and mouse studies suggests that cellular senescence contributes to the pathogenesis of AS. The senescent cells can be recognized by the irreversible loss of proliferative capacity, resistance to cell death, and the production of a bioactive secretome, known as the senescence-associated secretory phenotype (SASP). The main components of SASP include inflammatory cytokines, immune modulators, growth factors, and proteases (Childs et al., 2017). Recent findings in mouse models show that among cells with elevated p16INK4a and senescence-associated beta-galactosidase (SA-β-Gal) expression (common biomarkers of senescence) are Mφs (Prattichizzo et al., 2016; Liu et al., 2019). In mouse models of AS foamy senescent Mφs expressing inflammatory cytokines/chemokines accumulate in the subendothelial space in the early stages of disease while in advanced lesions there is evidence senescent Mφs promote plaque instability (Childs et al., 2016). Yang et al. found that microRNA-216a (miR-216a) promotes Mφ senescence as characterized by increased SA-β-GAL activity and p53 and p16 expression, and it has been reported that patients with vulnerable coronary plaques have elevated levels of plasma miR-216a (Yang et al., 2019). A recent study demonstrated that the senescent cells promote thinning of the fibrous cap, and the clearance of these cells by a synolitic agent (ABT263) helped maintain the fibrous caps in mice with advanced lesions. The senescent cells antagonize insulin-like growth factor (IGF)-1 through the secretion of IGF-binding protein-3, thereby inhibiting innate smooth muscle cell repair (Childs et al., 2021). Acting through the phosphatidylinositide 3 kinase-AKT-mTOR-p53 signal pathway, CD9 upregulation initiates cellular senescence while knocking it down in senescent cells reduces senescence (Brosseau et al., 2018; Cho et al., 2020). In human atherosclerotic lesions, most Mφs in plaques exhibited CD9 immunoreactivity (Nishida et al., 2000). In hyperlipidemic ApoE^−/−^ mice, selective delivery of the anti-senescence drug rosuvastatin to the atherosclerotic plaques using nanoparticles alleviated the progression of AS (Kim et al., 2021). Recent studies indicate that certain immune cells, in particular iNKT cells, can function to remove senescent cells (Kale et al., 2020; Arora et al., 2021).

## Macrophage-Based Therapeutic Strategies

Mφ dynamics play a role in all stages of AS and represent potential drug targets. A Mφ-biomimetic drug delivery system, in which ROS responsive nanoparticles were coated with Mφ membrane was developed. The Mφ membrane may sequester key pro-inflammatory cytokines to decrease local inflammation. The combination of pharmacotherapy and inflammatory cytokines sequestration offered by this platform led to improved therapeutic efficacy in AS (Gao et al., 2020).

Mφ death is an important feature of advanced plaques and is used as a therapeutic target in AS. Ox-LDL within advanced atherosclerotic plaque is directly responsible for the upregulation of RIP3, which is required to induce necroptosis in Mφs and lesion progression. Mφ necroptosis can be inhibited by necrostatin-1 to reduce lesion size and necrotic core formation for diagnostic and therapeutic interventions in AS (Karunakaran et al., 2016). Mφ pyroptosis is associated with the activation of NLRP3 inflammasome which has been linked to CV risk factors such as obesity and is an important regulator of CV inflammation. Thus, the therapeutic approaches targeting the activation of NLRP3 inflammasome and pyroptosis offer good prospects for the treatment of AS (Zeng et al., 2019). PARP inhibition attenuated plaque development and promoted plaque stability, likely through a reduction in the expression of inflammatory factors in ApoE^−/−^ mice. Thus, PARP1 inhibitors such as 3-aminobenzamide, INO-1001, methoxyflavones, PJ34, and DPQ may prove beneficial for the treatment of AS (Oumouna-Benachour et al., 2007; Martinet et al., 2019). Ferrostatin, an inhibitor of Mφ ferroptosis, suffers from inherent stability but anti-ferroptosis analog drugs with improved potency such as liproxstatins and anti-oxidants can inhibit ferroptotic cell death (Hofmans et al., 2016; Martinet et al., 2019).

Lipid deposition in the arterial wall is an initial step in AS, which promotes an inflammatory response. However, it was recently reported that the SR-B1 in ECs binds plasma LDL and mediates the delivery of LDL into arteries and its engulfment by artery wall Mφs to form foam cells. Thus, inhibition of SR-B1 in ECs might reduce lipid deposition with the potential of anti-inflammatory therapy in AS (Huang et al., 2019).

Photobiomodulation therapy has a protective role on AS through promoting the ABCA1-medicated cholesterol efflux in Mφ to inhibit foam cells formation (Yin et al., 2021). Proefferocytic therapy which specifically targets the necrotic core can potentially be used for the treatment of advanced AS (Kojima et al., 2017). 2-hydroxybenzylamine treatment reduced inflammation and plaque apoptotic cells but promoted efferocytosis and features of stable plaques in hypercholesterolemic *Ldlr* ^−/−^ mice supporting its potential as a therapeutic approach for atherosclerotic cardiovascular disease (Tao et al., 2020). An atheroprotective strategy that uses plaque targeting to deliver a proresolving mediator may stabilize advanced atherosclerotic lesions. Thus, defective inflammation resolution may have a role in advanced AS (Fredman et al., 2015).

Inhibition of local proliferation of Mφs is key in plaque regression in response to cholesterol-lowering. Thus, Mφ proliferation was identified as the predominant turnover determinant and a target for induction of plaque regression (Härdtner et al., 2020). HDL nanoparticles are used to target atherosclerotic Mφs. Nanoparticle-based delivery of simvastatin was used to inhibit plaque Mφ proliferation. This resulted in the rapid reduction of plaque inflammation when it was combined with oral statin treatment. Thus, pharmacologically inhibiting plaque Mφ proliferation by nanotherapy can effectively suppress plaque inflammation and reduce AS (Tang et al., 2015).

Defective autophagy in Mφs contributes to impaired cholesterol metabolism and defective efferocytosis leading. Mφs treated with anti-miR-33 showed increased autophagy, lipid droplet catabolism, and enhanced efferocytosis to reduce plaque necrosis (Ouimet et al., 2017).

## Conclusion and Future Perspective

AS is an inflammation-driven disease and Mφs play a central role in the pathogenesis of AS and controlling inflammation in all stages. Understanding monocytes differentiation into either pro- or anti-inflammatory Mφs within lesions and how Mφs affect plaque initiation and progression is of potential importance for the treatment of AS. Several strategies including anti-inflammatory approaches, depolarizing Mφs, modulation of Mφs, survival, and enhancing efferocytosis have been proposed as possible therapies. Targeting inflammation may be a promising therapeutic option for reducing AS. However, anti-inflammatory strategies may elicit undesirable effects such as infection (Moriya, 2019). Since the complex environmental stimuli within atherosclerotic plaques *in vivo* affects monocyte-derived Mφs, studying the effects of signaling crosstalk on inflammatory responses can be helpful to understand Mφ activation in the lesion and the development of therapeutic interventions. Another aspect that complicates therapeutic inhibition of Mφ death in atherosclerotic plaques is the crosstalk between cell death mechanisms (Nikoletopoulou et al., 2013). There is a balanced interaction between different types of cell death so that blocking one type of death may stimulate cells to initiate another death pathway. For instance, inhibition of caspases by the pan-caspase inhibitor zVAD promotes apoptosis but may facilitate the necroptosis program downstream of TNFR. Several main mediators of different types of cell death have been identified but more investigation is still needed (Chen et al., 2018). Cholesterol homeostasis in Mφs involves a dynamic balance between cholesterol uptake and efflux (Remmerie and Scott, 2018). These processes are regulated by signaling systems that are also affected by the events such as Mφ pro-inflammatory activation, phagocytosis stimulation, and autophagy induction. The signaling networks regulating these events in Mφs involve specific proteins that might be used as potential therapeutic targets in AS management. The divergent functions of Mφs, including contributions to inflammation, healing, regeneration, and remodeling arise due to the numerous types of Mφs, which can quickly adapt their phenotype in response to the microenvironment changes. Insufficient understanding of Mφ functions represents a major obstacle in Mφ targeting. For instance, M1 Mφs impair wound healing in some situations (Mirza et al., 2014) while promoting regeneration in others (Novak et al., 2014). M2 Mφs have also been associated with both tissue regeneration (Godwin et al., 2013) and fibrosis (Furukawa et al., 2015). In addition, data from cultured Mφs and animal models may not completely reflect the process in human atherosclerotic lesions. In this regard, Useful mouse models of plaque instability have been developed (Chen et al., 2013) Of course, translational studies are needed to confirm the observations made in preclinical murine models.

## References

[B1] Abdelaziz M. H. Abdelwahab S. F. Wan J. Cai W. Huixuan W. Jianjun C. (2020). Alternatively Activated Macrophages; a Double-Edged Sword in Allergic Asthma. J. Transl Med. 18 (1), 58. 10.1186/s12967-020-02251-w 32024540 PMC7003359

[B2] Alderson L. M. Endemann G. Lindsey S. Pronczuk A. Hoover R. L. Hayes K. C. (1986). LDL Enhances Monocyte Adhesion to Endothelial Cells *In Vitro* . Am. J. Pathol. 123 (2), 334–342. 3706494 PMC1888331

[B3] Apostolopoulos J. Davenport P. Tipping P. G. (1996). Interleukin-8 Production by Macrophages from Atheromatous Plaques. Arterioscler Thromb. Vasc. Biol. 16 (8), 1007–1012. 10.1161/01.atv.16.8.1007 8696939

[B4] Apostolov E. O. Shah S. V. Ray D. Basnakian A. G. (2009). Scavenger Receptors of Endothelial Cells Mediate the Uptake and Cellular Proatherogenic Effects of Carbamylated LDL. Arterioscler Thromb. Vasc. Biol. 29 (10), 1622–1630. 10.1161/atvbaha.109.189795 19696406 PMC5075391

[B5] Arango Duque G. Descoteaux A. (2014). Macrophage Cytokines: Involvement in Immunity and Infectious Diseases. Front. Immunol. 5, 491. 10.3389/fimmu.2014.00491 25339958 PMC4188125

[B6] Arora S. Thompson P. J. Wang Y. Bhattacharyya A. Apostolopoulou H. Hatano R. (2021). Invariant Natural Killer T Cells Coordinate Removal of Senescent Cells. Med 2 (8), 938–950.e8. 10.1016/j.medj.2021.04.014 34617070 PMC8491998

[B7] Arranz A. Doxaki C. Vergadi E. Martinez de la Torre Y. Vaporidi K. Lagoudaki E. D. (2012). Akt1 and Akt2 Protein Kinases Differentially Contribute to Macrophage Polarization. Proc. Natl. Acad. Sci. U S A. 109 (24), 9517–9522. 10.1073/pnas.1119038109 22647600 PMC3386059

[B8] Babaei S. Picard P. Ravandi A. Monge J. C. Lee T. C. Cernacek P. (2000). Blockade of Endothelin Receptors Markedly Reduces Atherosclerosis in LDL Receptor Deficient Mice: Role of Endothelin in Macrophage Foam Cell Formation. Cardiovasc. Res. 48 (1), 158–167. 10.1016/s0008-6363(00)00169-3 11033118

[B9] Babaev V. R. Hebron K. E. Wiese C. B. Toth C. L. Ding L. Zhang Y. (2014). Macrophage Deficiency of Akt2 Reduces Atherosclerosis in Ldlr Null Mice. J. Lipid Res. 55 (11), 2296–2308. 10.1194/jlr.M050633 25240046 PMC4617132

[B10] Barrett T. J. (2020). Macrophages in Atherosclerosis Regression. Atvb 40 (1), 20–33. 10.1161/atvbaha.119.312802 PMC6946104 31722535

[B11] Bartlett B. Ludewick H. P. Misra A. Lee S. Dwivedi G. (2019). Macrophages and T Cells in Atherosclerosis: a Translational Perspective. Am. J. Physiol. Heart Circ. Physiol. 317 (2), H375–H386. 10.1152/ajpheart.00206.2019 31199186

[B12] Bhaskar V. Yin J. Mirza A. M. Phan D. Vanegas S. Issafras H. (2011). Monoclonal Antibodies Targeting IL-1 Beta Reduce Biomarkers of Atherosclerosis *In Vitro* and Inhibit Atherosclerotic Plaque Formation in Apolipoprotein E-Deficient Mice. Atherosclerosis 216 (2), 313–320. 10.1016/j.atherosclerosis.2011.02.026 21411094

[B13] Bhat O. M. Kumar P. U. Giridharan N. V. Kaul D. Kumar M. J. Dhawan V. (2015). Interleukin-18-induced Atherosclerosis Involves CD36 and NF-Κb Crosstalk in Apo E-/- Mice. J. Cardiol. 66 (1), 28–35. 10.1016/j.jjcc.2014.10.012 25475966

[B14] Bi Y. Chen J. Hu F. Liu J. Li M. Zhao L. (2019). M2 Macrophages as a Potential Target for Antiatherosclerosis Treatment. Neural Plast. 2019, 6724903. 10.1155/2019/6724903 30923552 PMC6409015

[B15] Bick A. G. Pirruccello J. P. Griffin G. K. Gupta N. Gabriel S. Saleheen D. (2020). Genetic Interleukin 6 Signaling Deficiency Attenuates Cardiovascular Risk in Clonal Hematopoiesis. Circulation 141 (2), 124–131. 10.1161/CIRCULATIONAHA.119.044362 31707836 PMC7008855

[B16] Boada-Romero E. Martinez J. Heckmann B. L. Green D. R. (2020). The Clearance of Dead Cells by Efferocytosis. Nat. Rev. Mol. Cel Biol 21 (7), 398–414. 10.1038/s41580-020-0232-1 PMC7392086 32251387

[B17] Boesten L. S. Zadelaar A. S. van Nieuwkoop A. Gijbels M. J. de Winther M. P. Havekes L. M. (2005). Tumor Necrosis Factor-Alpha Promotes Atherosclerotic Lesion Progression in APOE*3-Leiden Transgenic Mice. Cardiovasc. Res. 66 (1), 179–185. 10.1016/j.cardiores.2005.01.001 15769461

[B18] Bosmans L. A. Bosch L. Kusters P. J. H. Lutgens E. Seijkens T. T. P. (2020). The CD40-Cd40l Dyad as Immunotherapeutic Target in Cardiovascular Disease. J. Cardiovasc. Transl Res. 14 (1), 1–10. 10.1007/s12265-020-09994-3 PMC7892683 32222950

[B19] Boyle J. J. Harrington H. A. Piper E. Elderfield K. Stark J. Landis R. C. (2009). Coronary Intraplaque Hemorrhage Evokes a Novel Atheroprotective Macrophage Phenotype. Am. J. Pathol. 174 (3), 1097–1108. 10.2353/ajpath.2009.080431 19234137 PMC2665768

[B20] Boyle J. J. (2005). Macrophage Activation in Atherosclerosis: Pathogenesis and Pharmacology of Plaque Rupture. Curr. Vasc. Pharmacol. 3 (1), 63–68. 10.2174/1570161052773861 15638783

[B21] Boyle J. J. Weissberg P. L. Bennett M. R. (2003). Tumor Necrosis Factor-Alpha Promotes Macrophage-Induced Vascular Smooth Muscle Cell Apoptosis by Direct and Autocrine Mechanisms. Arterioscler Thromb. Vasc. Biol. 23 (9), 1553–1558. 10.1161/01.atv.0000086961.44581.b7 12869351

[B22] Brosseau C. Colas L. Magnan A. Brouard S. (2018). CD9 Tetraspanin: a New Pathway for the Regulation of Inflammation? Front. Immunol. 9, 2316. 10.3389/fimmu.2018.02316 30356731 PMC6189363

[B23] Buscher K. Ehinger E. Gupta P. Pramod A. B. Wolf D. Tweet G. (2017). Natural Variation of Macrophage Activation as Disease-Relevant Phenotype Predictive of Inflammation and Cancer Survival. Nat. Commun. 8 (1), 16041–16110. 10.1038/ncomms16041 28737175 PMC5527282

[B24] Butcher M. J. Galkina E. V. (2012). Phenotypic and Functional Heterogeneity of Macrophages and Dendritic Cell Subsets in the Healthy and Atherosclerosis-Prone Aorta. Front. Physiol. 3, 44. 10.3389/fphys.2012.00044 22457649 PMC3307136

[B25] Canet-Soulas E. Bessueille L. Mechtouff L. Magne D. (2021). The Elusive Origin of Atherosclerotic Plaque Calcification. Front Cel Dev Biol 9, 622736. 10.3389/fcell.2021.622736 PMC7985066 33768090

[B26] Cardilo-Reis L. Gruber S. Schreier S. M. Drechsler M. Papac-Milicevic N. Weber C. (2012). Interleukin-13 Protects from Atherosclerosis and Modulates Plaque Composition by Skewing the Macrophage Phenotype. EMBO Mol. Med. 4 (10), 1072–1086. 10.1002/emmm.201201374 23027612 PMC3491837

[B27] Carmi Y. Voronov E. Dotan S. Lahat N. Rahat M. A. Fogel M. (2009). The Role of Macrophage-Derived IL-1 in Induction and Maintenance of Angiogenesis. J. Immunol. 183 (7), 4705–4714. 10.4049/jimmunol.0901511 19752225

[B28] Castrillo A. Joseph S. B. Vaidya S. A. Haberland M. Fogelman A. M. Cheng G. (2003). Crosstalk between LXR and Toll-like Receptor Signaling Mediates Bacterial and Viral Antagonism of Cholesterol Metabolism. Mol. Cel 12 (4), 805–816. 10.1016/s1097-2765(03)00384-8 14580333

[B29] Chan G. Bivins-Smith E. R. Smith M. S. Smith P. M. Yurochko A. D. (2008). Transcriptome Analysis Reveals Human Cytomegalovirus Reprograms Monocyte Differentiation toward an M1 Macrophage. J. Immunol. 181 (1), 698–711. 10.4049/jimmunol.181.1.698 18566437 PMC2614917

[B30] Chang H. Y. Lee H. N. Kim W. Surh Y. J. (2015). Docosahexaenoic Acid Induces M2 Macrophage Polarization through Peroxisome Proliferator-Activated Receptor γ Activation. Life Sci. 120, 39–47. 10.1016/j.lfs.2014.10.014 25445227

[B31] Chang T. Y. Li B. L. Chang C. C. Urano Y. (2009). Acyl-coenzyme A:cholesterol Acyltransferases. Am. J. Physiol. Endocrinol. Metab. 297 (1), E1–E9. 10.1152/ajpendo.90926.2008 19141679 PMC2711667

[B32] Chen P. Y. Qin L. Li G. Wang Z. Dahlman J. E. Malagon-Lopez J. (2019). Endothelial TGF-β Signalling Drives Vascular Inflammation and Atherosclerosis. Nat. Metab. 1 (9), 912–926. 10.1038/s42255-019-0102-3 31572976 PMC6767930

[B33] Chen Q. Kang J. Fu C. (2018). The independence of and Associations Among Apoptosis, Autophagy, and Necrosis. Signal. Transduct Target. Ther. 3 (1), 18–11. 10.1038/s41392-018-0018-5 29967689 PMC6026494

[B34] Chen Y. C. Bui A. V. Diesch J. Manasseh R. Hausding C. Rivera J. (2013). A Novel Mouse Model of Atherosclerotic Plaque Instability for Drug Testing and Mechanistic/therapeutic Discoveries Using Gene and microRNA Expression Profiling. Circ. Res. 113 (3), 252–265. 10.1161/CIRCRESAHA.113.301562 23748430

[B35] Cheng H. Wang Z. Fu L. Xu T. (2019). Macrophage Polarization in the Development and Progression of Ovarian Cancers: An Overview. Front. Oncol. 9, 421. 10.3389/fonc.2019.00421 31192126 PMC6540821

[B36] Chew M. Zhou J. Daugherty A. Eriksson T. Ellermann-Eriksen S. Hansen P. R. (2003). Thalidomide Inhibits Early Atherogenesis in apoE-Deficient Mice. APMIS Suppl. Suppl (109), 113–116. 12874961

[B37] Childs B. G. Baker D. J. Wijshake T. Conover C. A. Campisi J. Van Deursen J. M. (2016). Senescent Intimal Foam Cells Are Deleterious at All Stages of Atherosclerosis. Science 354 (6311), 472–477. 10.1126/science.aaf6659 27789842 PMC5112585

[B38] Childs B. G. Gluscevic M. Baker D. J. Laberge R. M. Marquess D. Dananberg J. (2017). Senescent Cells: an Emerging Target for Diseases of Ageing. Nat. Rev. Drug Discov. 16 (10), 718–735. 10.1038/nrd.2017.116 28729727 PMC5942225

[B39] Childs B. G. Zhang C. Shuja F. Sturmlechner I. Trewartha S. Velasco R. F. (2021). Senescent Cells Suppress Innate Smooth Muscle Cell Repair Functions in Atherosclerosis. Nat. Aging 1 (8), 1–17. 10.1038/s43587-021-00089-5 PMC8570576 34746803

[B40] Chinetti-Gbaguidi G. Colin S. Staels B. (2015). Macrophage Subsets in Atherosclerosis. Nat. Rev. Cardiol. 12 (1), 10–17. 10.1038/nrcardio.2014.173 25367649

[B41] Chistiakov D. A. Bobryshev Y. V. Orekhov A. N. (2015). Changes in Transcriptome of Macrophages in Atherosclerosis. J. Cel Mol Med 19 (6), 1163–1173. 10.1111/jcmm.12591 PMC4459832 25973901

[B42] Chistiakov D. A. Bobryshev Y. V. Orekhov A. N. (2016). Macrophage-mediated Cholesterol Handling in Atherosclerosis. J. Cel Mol Med 20 (1), 17–28. 10.1111/jcmm.12689 PMC4717859 26493158

[B43] Chistiakov D. A. Melnichenko A. A. Myasoedova V. A. Grechko A. V. Orekhov A. N. (2017). Mechanisms of Foam Cell Formation in Atherosclerosis. J. Mol. Med. (Berl) 95 (11), 1153–1165. 10.1007/s00109-017-1575-8 28785870

[B44] Cho J. H. Kim E. C. Son Y. Lee D. W. Park Y. S. Choi J. H. (2020). CD9 Induces Cellular Senescence and Aggravates Atherosclerotic Plaque Formation. Cell Death Differ 27 (9), 2681–2696. 10.1038/s41418-020-0537-9 32346137 PMC7429960

[B45] Coats B. R. Schoenfelt K. Q. Barbosa-Lorenzi V. C. Peris E. Cui C. Hoffman A. (2017). Metabolically Activated Adipose Tissue Macrophages Perform Detrimental and Beneficial Functions during Diet-Induced Obesity. Cell Rep 20 (13), 3149–3161. 10.1016/j.celrep.2017.08.096 28954231 PMC5646237

[B46] Cochain C. Vafadarnejad E. Arampatzi P. Pelisek J. Winkels H. Ley K. (2018). Single-cell RNA-Seq Reveals the Transcriptional Landscape and Heterogeneity of Aortic Macrophages in Murine Atherosclerosis. Circ. Res. 122 (12), 1661–1674. 10.1161/CIRCRESAHA.117.312509 29545365

[B47] Colin S. Chinetti-Gbaguidi G. Staels B. (2014). Macrophage Phenotypes in Atherosclerosis. Immunol. Rev. 262 (1), 153–166. 10.1111/imr.12218 25319333

[B48] Covarrubias A. J. Aksoylar H. I. Horng T. (2015). “Control of Macrophage Metabolism and Activation by mTOR and Akt Signaling,” in Seminars in Immunology (Elsevier), 286–296. 10.1016/j.smim.2015.08.001 PMC4682888 26360589

[B49] Davenport P. Tipping P. G. (2003). The Role of Interleukin-4 and Interleukin-12 in the Progression of Atherosclerosis in Apolipoprotein E-Deficient Mice. Am. J. Pathol. 163 (3), 1117–1125. 10.1016/s0002-9440(10)63471-2 12937153 PMC1868277

[B50] de Jager S. C. Bermúdez B. Bot I. Koenen R. R. Bot M. Kavelaars A. (2011). Growth Differentiation Factor 15 Deficiency Protects against Atherosclerosis by Attenuating CCR2-Mediated Macrophage Chemotaxis. J. Exp. Med. 208 (2), 217–225. 10.1084/jem.20100370 21242297 PMC3039852

[B51] De Paoli F. Staels B. Chinetti-Gbaguidi G. (2014). Macrophage Phenotypes and Their Modulation in Atherosclerosis. Circ. J. 78 (8), 1775–1781. 10.1253/circj.cj-14-0621 24998279

[B52] Deczkowska A. Weiner A. Amit I. (2020). The Physiology, Pathology, and Potential Therapeutic Applications of the TREM2 Signaling Pathway. Cell 181 (6), 1207–1217. 10.1016/j.cell.2020.05.003 32531244

[B53] Devries-Seimon T. Li Y. Yao P. M. Stone E. Wang Y. Davis R. J. (2005). Cholesterol-induced Macrophage Apoptosis Requires ER Stress Pathways and Engagement of the Type A Scavenger Receptor. J. Cel Biol 171 (1), 61–73. 10.1083/jcb.200502078 PMC2171235 16203857

[B54] Dinarello C. A. Novick D. Kim S. Kaplanski G. (2013). Interleukin-18 and IL-18 Binding Protein. Front. Immunol. 4, 289. 10.3389/fimmu.2013.00289 24115947 PMC3792554

[B55] Douna H. Amersfoort J. Schaftenaar F. H. Kroon S. van Puijvelde G. H. M. Kuiper J. (2019). Bidirectional Effects of IL-10+ Regulatory B Cells in Ldlr-/- Mice. Atherosclerosis 280, 118–125. 10.1016/j.atherosclerosis.2018.11.019 30500604

[B56] Erbilgin A. Seldin M. M. Wu X. Mehrabian M. Zhou Z. Qi H. (2018). Transcription Factor Zhx2 Deficiency Reduces Atherosclerosis and Promotes Macrophage Apoptosis in Mice. Arterioscler Thromb. Vasc. Biol. 38 (9), 2016–2027. 10.1161/ATVBAHA.118.311266 30026271 PMC6202168

[B57] Febbraio M. Hajjar D. P. Silverstein R. L. (2001). CD36: a Class B Scavenger Receptor Involved in Angiogenesis, Atherosclerosis, Inflammation, and Lipid Metabolism. J. Clin. Invest. 108 (6), 785–791. 10.1172/jci14006 11560944 PMC200943

[B58] Feig J. E. Parathath S. Rong J. X. Mick S. L. Vengrenyuk Y. Grauer L. (2011). Reversal of Hyperlipidemia with a Genetic Switch Favorably Affects the Content and Inflammatory State of Macrophages in Atherosclerotic Plaques. Circulation 123 (9), 989–998. 10.1161/circulationaha.110.984146 21339485 PMC3131163

[B59] Fernández-Hernando C. Ackah E. Yu J. Suárez Y. Murata T. Iwakiri Y. (2007). Loss of Akt1 Leads to Severe Atherosclerosis and Occlusive Coronary Artery Disease. Cell Metab 6 (6), 446–457. 10.1016/j.cmet.2007.10.007 18054314 PMC3621848

[B60] Fernandez D. M. Rahman A. H. Fernandez N. F. Chudnovskiy A. Amir E. D. Amadori L. (2019). Single-cell Immune Landscape of Human Atherosclerotic Plaques. Nat. Med. 25 (10), 1576–1588. 10.1038/s41591-019-0590-4 31591603 PMC7318784

[B61] Fernandez D. M. Giannarelli C. (2021). Immune Cell Profiling in Atherosclerosis: Role in Research and Precision Medicine. Nat. Rev. Cardiol., 1–16. 10.1038/s41569-021-00589-2 34267377 PMC8280607

[B62] Fidler T. P. Xue C. Yalcinkaya M. Hardaway B. Abramowicz S. Xiao T. (2021). The AIM2 Inflammasome Exacerbates Atherosclerosis in Clonal Haematopoiesis. Nature 592 (7853), 296–301. 10.1038/s41586-021-03341-5 33731931 PMC8038646

[B63] Fourman L. T. Saylor C. F. Cheru L. Fitch K. Looby S. Keller K. (2020). Anti-Inflammatory Interleukin 10 Inversely Relates to Coronary Atherosclerosis in Persons with Human Immunodeficiency Virus. J. Infect. Dis. 221 (4), 510–515. 10.1093/infdis/jiz254 31077265 PMC7325621

[B64] Frank D. Vince J. E. (2019). Pyroptosis versus Necroptosis: Similarities, Differences, and Crosstalk. Cel Death Differ 26 (1), 99–114. 10.1038/s41418-018-0212-6 PMC6294779 30341423

[B65] Fredman G. Kamaly N. Spolitu S. Milton J. Ghorpade D. Chiasson R. (2015). Erratum for the Research Article: "Targeted Nanoparticles Containing the Proresolving Peptide Ac2-26 Protect against Advanced Atherosclerosis in Hypercholesterolemic Mice" by G. Fredman, N. Kamaly, S. Spolitu, J. Milton, D. Ghorpade, R. Chiasson, G. Kuriakose, M. Perretti, O. Farokzhad, I. Tabas. Sci. Transl Med. 7 (275), 277er2-275ra220. 10.1126/scitranslmed.aaa106510.1126/scitranslmed.aaa9877 PMC4397585 25695999

[B66] Furukawa S. Moriyama M. Tanaka A. Maehara T. Tsuboi H. Iizuka M. (2015). Preferential M2 Macrophages Contribute to Fibrosis in IgG4-Related Dacryoadenitis and Sialoadenitis, So-Called Mikulicz's Disease. Clin. Immunol. 156 (1), 9–18. 10.1016/j.clim.2014.10.008 25450336

[B67] Fuster J. J. MacLauchlan S. Zuriaga M. A. Polackal M. N. Ostriker A. C. Chakraborty R. (2017). Clonal Hematopoiesis Associated with TET2 Deficiency Accelerates Atherosclerosis Development in Mice. Science 355 (6327), 842–847. 10.1126/science.aag1381 28104796 PMC5542057

[B68] Gao C. Huang Q. Liu C. Kwong C. H. T. Yue L. Wan J. B. (2020). Treatment of Atherosclerosis by Macrophage-Biomimetic Nanoparticles via Targeted Pharmacotherapy and Sequestration of Proinflammatory Cytokines. Nat. Commun. 11 (1), 2622–2714. 10.1038/s41467-020-16439-7 32457361 PMC7251120

[B69] Gerrick K. Y. Gerrick E. R. Gupta A. Wheelan S. J. Yegnasubramanian S. Jaffee E. M. (2018). Transcriptional Profiling Identifies Novel Regulators of Macrophage Polarization. PLoS One 13 (12), e0208602. 10.1371/journal.pone.0208602 30532146 PMC6286176

[B70] Gerszten R. E. Garcia-Zepeda E. A. Lim Y. C. Yoshida M. Ding H. A. Gimbrone M. A. (1999). MCP-1 and IL-8 Trigger Firm Adhesion of Monocytes to Vascular Endothelium under Flow Conditions. Nature 398 (6729), 718–723. 10.1038/19546 10227295

[B71] Giacona M. B. Papapanou P. N. Lamster I. B. Rong L. L. D'Agati V. D. Schmidt A. M. (2004). Porphyromonas Gingivalis Induces its Uptake by Human Macrophages and Promotes Foam Cell Formation *In Vitro* . FEMS Microbiol. Lett. 241 (1), 95–101. 10.1016/j.femsle.2004.10.009 15556715

[B72] Godwin J. W. Pinto A. R. Rosenthal N. A. (2013). Macrophages Are Required for Adult Salamander Limb Regeneration. Proc. Natl. Acad. Sci. U S A. 110 (23), 9415–9420. 10.1073/pnas.1300290110 23690624 PMC3677454

[B73] Gong D. Shi W. Yi S. J. Chen H. Groffen J. Heisterkamp N. (2012). TGFβ Signaling Plays a Critical Role in Promoting Alternative Macrophage Activation. BMC Immunol. 13 (1), 31–10. 10.1186/1471-2172-13-31 22703233 PMC3406960

[B74] Gonzalez L. Trigatti B. L. (2017). Macrophage Apoptosis and Necrotic Core Development in Atherosclerosis: a Rapidly Advancing Field with Clinical Relevance to Imaging and Therapy. Can. J. Cardiol. 33 (3), 303–312. 10.1016/j.cjca.2016.12.010 28232016

[B75] Goossens P. Gijbels M. J. Zernecke A. Eijgelaar W. Vergouwe M. N. van der Made I. (2010). Myeloid Type I Interferon Signaling Promotes Atherosclerosis by Stimulating Macrophage Recruitment to Lesions. Cel Metab 12 (2), 142–153. 10.1016/j.cmet.2010.06.008 20674859

[B76] Gough P. J. Gomez I. G. Wille P. T. Raines E. W. (2006). Macrophage Expression of Active MMP-9 Induces Acute Plaque Disruption in apoE-Deficient Mice. J. Clin. Invest. 116 (1), 59–69. 10.1172/JCI25074 16374516 PMC1319218

[B77] Grainger D. J. Kemp P. R. Metcalfe J. C. Liu A. C. Lawn R. M. Williams N. R. (1995). The Serum Concentration of Active Transforming Growth Factor-Beta Is Severely Depressed in Advanced Atherosclerosis. Nat. Med. 1 (1), 74–79. 10.1038/nm0195-74 7584958

[B78] Guo Z. Ran Q. Roberts L. J. II Zhou L. Richardson A. Sharan C. (2008). Suppression of Atherogenesis by Overexpression of Glutathione Peroxidase-4 in Apolipoprotein E-Deficient Mice. Free Radic. Biol. Med. 44 (3), 343–352. 10.1016/j.freeradbiomed.2007.09.009 18215741 PMC2245803

[B79] Härdtner C. Kornemann J. Krebs K. Ehlert C. A. Jander A. Zou J. (2020). Inhibition of Macrophage Proliferation Dominates Plaque Regression in Response to Cholesterol Lowering. Basic Res. Cardiol. 115 (6), 78–19. 10.1007/s00395-020-00838-4 33296022 PMC7725697

[B80] Habib A. Finn A. V. (2014). The Role of Iron Metabolism as a Mediator of Macrophage Inflammation and Lipid Handling in Atherosclerosis. Front. Pharmacol. 5, 195. 10.3389/fphar.2014.00195 25221512 PMC4145350

[B81] Haka A. S. Barbosa-Lorenzi V. C. Lee H. J. Falcone D. J. Hudis C. A. Dannenberg A. J. (2016). Exocytosis of Macrophage Lysosomes Leads to Digestion of Apoptotic Adipocytes and Foam Cell Formation. J. Lipid Res. 57 (6), 980–992. 10.1194/jlr.M064089 27044658 PMC4878183

[B82] Henson P. M. Bratton D. L. Fadok V. A. (2001). Apoptotic Cell Removal. Curr. Biol. 11 (19), R795–R805. 10.1016/s0960-9822(01)00474-2 11591341

[B83] Heyde A. Rohde D. McAlpine C. S. Zhang S. Hoyer F. F. Gerold J. M. (2021). Increased Stem Cell Proliferation in Atherosclerosis Accelerates Clonal Hematopoiesis. Cell 184 (5), 1348–e22. e1322. 10.1016/j.cell.2021.01.049 33636128 PMC8109274

[B84] Hoebe K. Georgel P. Rutschmann S. Du X. Mudd S. Crozat K. (2005). CD36 Is a Sensor of Diacylglycerides. Nature 433 (7025), 523–527. 10.1038/nature03253 15690042

[B85] Hofmans S. Vanden Berghe T. Devisscher L. Hassannia B. Lyssens S. Joossens J. (2016). Novel Ferroptosis Inhibitors with Improved Potency and ADME Properties. J. Med. Chem. 59 (5), 2041–2053. 10.1021/acs.jmedchem.5b01641 26696014

[B86] Huang L. Chambliss K. L. Gao X. Yuhanna I. S. Behling-Kelly E. Bergaya S. (2019). SR-B1 Drives Endothelial Cell LDL Transcytosis via DOCK4 to Promote Atherosclerosis. Nature 569 (7757), 565–569. 10.1038/s41586-019-1140-4 31019307 PMC6631346

[B87] Huo Y. Xia L. (2009). P-selectin Glycoprotein Ligand-1 Plays a Crucial Role in the Selective Recruitment of Leukocytes into the Atherosclerotic Arterial wall. Trends Cardiovasc. Med. 19 (4), 140–145. 10.1016/j.tcm.2009.07.006 19818951 PMC2762112

[B88] Jaiswal S. Natarajan P. Silver A. J. Gibson C. J. Bick A. G. Shvartz E. (2017). Clonal Hematopoiesis and Risk of Atherosclerotic Cardiovascular Disease. N. Engl. J. Med. 377 (2), 111–121. 10.1056/NEJMoa1701719 28636844 PMC6717509

[B89] Jansen M. F. Hollander M. R. van Royen N. Horrevoets A. J. Lutgens E. (2016). CD40 in Coronary Artery Disease: a Matter of Macrophages? Basic Res. Cardiol. 111 (4), 38. 10.1007/s00395-016-0554-5 27146510 PMC4856717

[B90] Jefferis B. J. Papacosta O. Owen C. G. Wannamethee S. G. Humphries S. E. Woodward M. (2011). Interleukin 18 and Coronary Heart Disease: Prospective Study and Systematic Review. Atherosclerosis 217 (1), 227–233. 10.1016/j.atherosclerosis.2011.03.015 21481392 PMC3146704

[B91] Jiang Y. Gao Q. Wang L. Guo C. Zhu F. Wang B. (2016). Deficiency of Programmed Cell Death 4 Results in Increased IL-10 Expression by Macrophages and Thereby Attenuates Atherosclerosis in Hyperlipidemic Mice. Cell Mol Immunol 13 (4), 524–534. 10.1038/cmi.2015.47 26166769 PMC4947820

[B92] Jung S. B. Choi M. J. Ryu D. Yi H. S. Lee S. E. Chang J. Y. (2018). Reduced Oxidative Capacity in Macrophages Results in Systemic Insulin Resistance. Nat. Commun. 9 (1), 1551–1615. 10.1038/s41467-018-03998-z 29674655 PMC5908799

[B93] Kadl A. Meher A. K. Sharma P. R. Lee M. Y. Doran A. C. Johnstone S. R. (2010). Identification of a Novel Macrophage Phenotype that Develops in Response to Atherogenic Phospholipids via Nrf2. Circ. Res. 107 (6), 737–746. 10.1161/circresaha.109.215715 20651288 PMC2941538

[B94] Kale A. Sharma A. Stolzing A. Desprez P. Y. Campisi J. (2020). Role of Immune Cells in the Removal of Deleterious Senescent Cells. Immun. Ageing 17 (1), 16–19. 10.1186/s12979-020-00187-9 32518575 PMC7271494

[B95] Kamari Y. Shaish A. Shemesh S. Vax E. Grosskopf I. Dotan S. (2011). Reduced Atherosclerosis and Inflammatory Cytokines in Apolipoprotein-E-Deficient Mice Lacking Bone Marrow-Derived Interleukin-1α. Biochem. Biophys. Res. Commun. 405 (2), 197–203. 10.1016/j.bbrc.2011.01.008 21219852

[B96] Kapellos T. S. Bonaguro L. Gemünd I. Reusch N. Saglam A. Hinkley E. R. (2019). Human Monocyte Subsets and Phenotypes in Major Chronic Inflammatory Diseases. Front. Immunol. 10, 2035. 10.3389/fimmu.2019.02035 31543877 PMC6728754

[B97] Kaperonis E. A. Liapis C. D. Kakisis J. D. Dimitroulis D. Papavassiliou V. G. (2006). Inflammation and Atherosclerosis. Eur. J. Vasc. Endovasc Surg. 31 (4), 386–393. 10.1016/j.ejvs.2005.11.001 16359887

[B98] Karunakaran D. Geoffrion M. Wei L. Gan W. Richards L. Shangari P. (2016). Targeting Macrophage Necroptosis for Therapeutic and Diagnostic Interventions in Atherosclerosis. Sci. Adv. 2 (7), e1600224. 10.1126/sciadv.1600224 27532042 PMC4985228

[B99] Kattoor A. J. Goel A. Mehta J. L. (2019). LOX-1: Regulation, Signaling and its Role in Atherosclerosis. Antioxidants (Basel) 8 (7), 218. 10.3390/antiox8070218 PMC6680802 31336709

[B100] Khallou-Laschet J. Varthaman A. Fornasa G. Compain C. Gaston A. T. Clement M. (2010). Macrophage Plasticity in Experimental Atherosclerosis. PLoS One 5 (1), e8852. 10.1371/journal.pone.0008852 20111605 PMC2810335

[B101] Kim K. Shim D. Lee J. S. Zaitsev K. Williams J. W. Kim K. W. (2018). Transcriptome Analysis Reveals Nonfoamy rather Than Foamy Plaque Macrophages Are Proinflammatory in Atherosclerotic Murine Models. Circ. Res. 123 (10), 1127–1142. 10.1161/CIRCRESAHA.118.312804 30359200 PMC6945121

[B102] Kinlay S. Schwartz G. G. Olsson A. G. Rifai N. Szarek M. Waters D. D. (2008). Inflammation, Statin Therapy, and Risk of Stroke after an Acute Coronary Syndrome in the MIRACL Study. Arterioscler Thromb. Vasc. Biol. 28 (1), 142–147. 10.1161/ATVBAHA.107.151787 17991875

[B103] Kleemann R. Zadelaar S. Kooistra T. (2008). Cytokines and Atherosclerosis: a Comprehensive Review of Studies in Mice. Cardiovasc. Res. 79 (3), 360–376. 10.1093/cvr/cvn120 18487233 PMC2492729

[B104] Kober D. L. Brett T. J. (2017). TREM2-Ligand Interactions in Health and Disease. J. Mol. Biol. 429 (11), 1607–1629. 10.1016/j.jmb.2017.04.004 28432014 PMC5485854

[B105] Kojima Y. Volkmer J. P. McKenna K. Civelek M. Lusis A. J. Miller C. L. (2016). CD47-blocking Antibodies Restore Phagocytosis and Prevent Atherosclerosis. Nature 536 (7614), 86–90. 10.1038/nature18935 27437576 PMC4980260

[B106] Kojima Y. Weissman I. L. Leeper N. J. (2017). The Role of Efferocytosis in Atherosclerosis. Circulation 135 (5), 476–489. 10.1161/circulationaha.116.025684 28137963 PMC5302553

[B107] Kolter J. Kierdorf K. Henneke P. (2020a). Origin and Differentiation of Nerve-Associated Macrophages. J. Immunol. 204 (2), 271–279. 10.4049/jimmunol.1901077 31907269

[B108] Kolter J. Kierdorf K. Henneke P. (2020b). Origin and Differentiation of Nerve-Associated Macrophages. J. Immunol. 204 (2), 271–279. 10.4049/jimmunol.1901077 31907269

[B109] Kozarsky K. F. Donahee M. H. Rigotti A. Iqbal S. N. Edelman E. R. Krieger M. (1997). Overexpression of the HDL Receptor SR-BI Alters Plasma HDL and Bile Cholesterol Levels. Nature 387 (6631), 414–417. 10.1038/387414a0 9163428

[B110] Lehtolainen P. Takeya M. Ylä-Herttuala S. (2000). Retrovirus-mediated, Stable Scavenger-Receptor Gene Transfer Leads to Functional Endocytotic Receptor Expression, Foam Cell Formation, and Increased Susceptibility to Apoptosis in Rabbit Aortic Smooth Muscle Cells. Arterioscler Thromb. Vasc. Biol. 20 (1), 52–60. 10.1161/01.atv.20.1.52 10634800

[B111] Leitinger N. Schulman I. G. (2013). Phenotypic Polarization of Macrophages in Atherosclerosis. Arterioscler Thromb. Vasc. Biol. 33 (6), 1120–1126. 10.1161/ATVBAHA.112.300173 23640492 PMC3745999

[B112] Leuschner F. Rauch P. J. Ueno T. Gorbatov R. Marinelli B. Lee W. W. (2012). Rapid Monocyte Kinetics in Acute Myocardial Infarction Are Sustained by Extramedullary Monocytopoiesis. J. Exp. Med. 209 (1), 123–137. 10.1084/jem.20111009 22213805 PMC3260875

[B113] Ley K. Laudanna C. Cybulsky M. I. Nourshargh S. (2007). Getting to the Site of Inflammation: the Leukocyte Adhesion cascade Updated. Nat. Rev. Immunol. 7 (9), 678–689. 10.1038/nri2156 17717539

[B114] Li B. Xia Y. Hu B. (2020a). Infection and Atherosclerosis: TLR-dependent Pathways. Cell Mol Life Sci 77 (14), 2751–2769. 10.1007/s00018-020-03453-7 32002588 PMC7223178

[B115] Li Y. Yun K. Mu R. (2020b). A Review on the Biology and Properties of Adipose Tissue Macrophages Involved in Adipose Tissue Physiological and Pathophysiological Processes. Lipids Health Dis. 19 (1), 164–169. 10.1186/s12944-020-01342-3 32646451 PMC7350193

[B116] Libby P. (2021). The Changing Landscape of Atherosclerosis. Nature 592 (7855), 524–533. 10.1038/s41586-021-03392-8 33883728

[B117] Liberale L. Dallegri F. Montecucco F. Carbone F. (2017). Pathophysiological Relevance of Macrophage Subsets in Atherogenesis. Thromb. Haemost. 117 (01), 7–18. 10.1160/TH16-08-0593 27683760

[B118] Lim W. S. Timmins J. M. Seimon T. A. Sadler A. Kolodgie F. D. Virmani R. (2008). Signal Transducer and Activator of Transcription-1 Is Critical for Apoptosis in Macrophages Subjected to Endoplasmic Reticulum Stress *In Vitro* and in Advanced Atherosclerotic Lesions *In Vivo* . Circulation 117 (7), 940–951. 10.1161/circulationaha.107.711275 18227389 PMC2276635

[B119] Lin J. D. Nishi H. Poles J. Niu X. Mccauley C. Rahman K. (2019). Single-cell Analysis of Fate-Mapped Macrophages Reveals Heterogeneity, Including Stem-like Properties, during Atherosclerosis Progression and Regression. JCI insight 4 (4). 10.1172/jci.insight.124574 PMC6478411 30830865

[B120] Lin P. Ji H.-H. Li Y.-J. Guo S.-D. (2021). Macrophage Plasticity and Atherosclerosis Therapy. Front. Mol. Biosciences 8, 324. 10.3389/fmolb.2021.679797 PMC8138136 34026849

[B121] Liu J. Y. Souroullas G. P. Diekman B. O. Krishnamurthy J. Hall B. M. Sorrentino J. A. (2019). Cells Exhibiting strong P16 INK4a Promoter Activation *In Vivo* Display Features of Senescence. Proc. Natl. Acad. Sci. U S A. 116 (7), 2603–2611. 10.1073/pnas.1818313116 30683717 PMC6377452

[B122] Low E. L. Baker A. H. Bradshaw A. C. (2019). TGFβ, Smooth Muscle Cells and Coronary Artery Disease: a Review. Cell Signal 53, 90–101. 10.1016/j.cellsig.2018.09.004 30227237 PMC6293316

[B123] Mallat Z. Gojova A. Marchiol-Fournigault C. Esposito B. Kamaté C. Merval R. (2001). Inhibition of Transforming Growth Factor-Beta Signaling Accelerates Atherosclerosis and Induces an Unstable Plaque Phenotype in Mice. Circ. Res. 89 (10), 930–934. 10.1161/hh2201.099415 11701621

[B124] Mallat Z. Heymes C. Ohan J. Faggin E. Lesèche G. Tedgui A. (1999). Expression of Interleukin-10 in Advanced Human Atherosclerotic Plaques: Relation to Inducible Nitric Oxide Synthase Expression and Cell Death. Arterioscler Thromb. Vasc. Biol. 19 (3), 611–616. 10.1161/01.atv.19.3.611 10073964

[B125] Manning-Tobin J. J. Moore K. J. Seimon T. A. Bell S. A. Sharuk M. Alvarez-Leite J. I. (2009). Loss of SR-A and CD36 Activity Reduces Atherosclerotic Lesion Complexity without Abrogating Foam Cell Formation in Hyperlipidemic Mice. Arterioscler Thromb. Vasc. Biol. 29 (1), 19–26. 10.1161/atvbaha.108.176644 18948635 PMC2666043

[B126] Mantovani A. Sica A. Sozzani S. Allavena P. Vecchi A. Locati M. (2004). The Chemokine System in Diverse Forms of Macrophage Activation and Polarization. Trends Immunol. 25 (12), 677–686. 10.1016/j.it.2004.09.015 15530839

[B127] Marchio P. Guerra-Ojeda S. Vila J. M. Aldasoro M. Victor V. M. Mauricio M. D. (2019). Targeting Early Atherosclerosis: A Focus on Oxidative Stress and Inflammation. *Oxidative Medicine and Cellular Longevity* 2019. 10.1155/2019/8563845 PMC6636482 31354915

[B128] Martinet W. Coornaert I. Puylaert P. De Meyer G. R. Y. (2019). Macrophage Death as a Pharmacological Target in Atherosclerosis. Front. Pharmacol. 10, 306. 10.3389/fphar.2019.00306 31019462 PMC6458279

[B129] McAlpine C. S. Kiss M. G. Rattik S. He S. Vassalli A. Valet C. (2019). Sleep Modulates Haematopoiesis and Protects against Atherosclerosis. Nature 566 (7744), 383–387. 10.1038/s41586-019-0948-2 30760925 PMC6442744

[B130] McKellar G. E. McCarey D. W. Sattar N. McInnes I. B. (2009). Role for TNF in Atherosclerosis? Lessons from Autoimmune Disease. Nat. Rev. Cardiol. 6 (6), 410–7. 10.1038/nrcardio.2009.57 19421244

[B131] Medbury H. J. Williams H. Fletcher J. P. (2014). Clinical Significance of Macrophage Phenotypes in Cardiovascular Disease. Clin. Transl Med. 3 (1), 63–10. 10.1186/s40169-014-0042-1 25635207 PMC4303745

[B132] Meng Z. Wang M. Xing J. Liu Y. Li H. (2019). Myricetin Ameliorates Atherosclerosis in the Low-Density-Lipoprotein Receptor Knockout Mice by Suppression of Cholesterol Accumulation in Macrophage Foam Cells. Nutr. Metab. (Lond) 16 (1), 25–29. 10.1186/s12986-019-0354-7 31049071 PMC6482568

[B133] Michelsen K. S. Wong M. H. Shah P. K. Zhang W. Yano J. Doherty T. M. (2004). Lack of Toll-like Receptor 4 or Myeloid Differentiation Factor 88 Reduces Atherosclerosis and Alters Plaque Phenotype in Mice Deficient in Apolipoprotein E. Proc. Natl. Acad. Sci. U S A. 101 (29), 10679–10684. 10.1073/pnas.0403249101 15249654 PMC489994

[B134] Miki H. Pei H. Gracias D. T. Linden J. Croft M. (2021). Clearance of Apoptotic Cells by Lung Alveolar Macrophages Prevents Development of House Dust Mite-Induced Asthmatic Lung Inflammation. J. Allergy Clin. Immunol. 147 (3), 1087–1092. 10.1016/j.jaci.2020.10.005 33065121 PMC7940554

[B135] Milioti N. Bermudez-Fajardo A. Penichet M. L. Oviedo-Orta E. (2008). Antigen-induced Immunomodulation in the Pathogenesis of Atherosclerosis. Clin. Dev. Immunol. 2008, 723539. 10.1155/2008/723539 18551190 PMC2423423

[B136] Mirza R. E. Fang M. M. Weinheimer-Haus E. M. Ennis W. J. Koh T. J. (2014). Sustained Inflammasome Activity in Macrophages Impairs Wound Healing in Type 2 Diabetic Humans and Mice. Diabetes 63 (3), 1103–1114. 10.2337/db13-0927 24194505 PMC3931398

[B137] Moore K. J. Sheedy F. J. Fisher E. A. (2013). Macrophages in Atherosclerosis: a Dynamic Balance. Nat. Rev. Immunol. 13 (10), 709–721. 10.1038/nri3520 23995626 PMC4357520

[B138] Moreno Velásquez I. Gajulapuri A. Leander K. Berglund A. de Faire U. Gigante B. (2019). Serum IL8 Is Not Associated with Cardiovascular Events but with All-Cause Mortality. BMC Cardiovasc. Disord. 19 (1), 34–38. 10.1186/s12872-019-1014-6 30717657 PMC6360748

[B139] Moriya J. (2019). Critical Roles of Inflammation in Atherosclerosis. J. Cardiol. 73 (1), 22–27. 10.1016/j.jjcc.2018.05.010 29907363

[B140] Murray P. J. (2017). Macrophage Polarization. Annu. Rev. Physiol. 79, 541–566. 10.1146/annurev-physiol-022516-034339 27813830

[B141] Nagenborg J. Goossens P. Biessen E. A. L. Donners M. M. P. C. (2017). Heterogeneity of Atherosclerotic Plaque Macrophage Origin, Phenotype and Functions: Implications for Treatment. Eur. J. Pharmacol. 816, 14–24. 10.1016/j.ejphar.2017.10.005 28989084

[B142] Nahrendorf M. Swirski F. K. Aikawa E. Stangenberg L. Wurdinger T. Figueiredo J. L. (2007). The Healing Myocardium Sequentially Mobilizes Two Monocyte Subsets with Divergent and Complementary Functions. J. Exp. Med. 204 (12), 3037–3047. 10.1084/jem.20070885 18025128 PMC2118517

[B143] Neufeld E. B. O'Brien K. Walts A. D. Stonik J. A. Demosky S. J. Malide D. (2014). Cellular Localization and Trafficking of the Human ABCG1 Transporter. Biology (Basel) 3 (4), 781–800. 10.3390/biology3040781 25405320 PMC4280511

[B144] Nikoletopoulou V. Markaki M. Palikaras K. Tavernarakis N. (2013). Crosstalk between Apoptosis, Necrosis and Autophagy. Biochim. Biophys. Acta 1833 (12), 3448–3459. 10.1016/j.bbamcr.2013.06.001 23770045

[B145] Nikonova A. Khaitov M. Jackson D. J. Traub S. Trujillo-Torralbo M. B. Kudlay D. A. (2020). M1-like Macrophages Are Potent Producers of Anti-viral Interferons and M1-Associated Marker-Positive Lung Macrophages Are Decreased during Rhinovirus-Induced Asthma Exacerbations. EBioMedicine 54, 102734. 10.1016/j.ebiom.2020.102734 32279057 PMC7152663

[B146] Nilchian A. Plant E. Parniewska M. M. Santiago A. Rossignoli A. Skogsberg J. (2020). Induction of the Coxsackievirus and Adenovirus Receptor in Macrophages during the Formation of Atherosclerotic Plaques. J. Infect. Dis. 222 (12), 2041–2051. 10.1093/infdis/jiaa418 32852032 PMC7661765

[B147] Nishida M. Miyagawa J. Yamashita S. Higashiyama S. Nakata A. Ouchi N. (2000). Localization of CD9, an Enhancer Protein for Proheparin-Binding Epidermal Growth Factor-like Growth Factor, in Human Atherosclerotic Plaques: Possible Involvement of Juxtacrine Growth Mechanism on Smooth Muscle Cell Proliferation. Arterioscler Thromb. Vasc. Biol. 20 (5), 1236–1243. 10.1161/01.atv.20.5.1236 10807738

[B148] Novak M. L. Weinheimer-Haus E. M. Koh T. J. (2014). Macrophage Activation and Skeletal Muscle Healing Following Traumatic Injury. J. Pathol. 232 (3), 344–355. 10.1002/path.4301 24255005 PMC4019602

[B149] Ohgami N. Nagai R. Ikemoto M. Arai H. Kuniyasu A. Horiuchi S. (2001). Cd36, a Member of the Class B Scavenger Receptor Family, as a Receptor for Advanced Glycation End Products. J. Biol. Chem. 276 (5), 3195–3202. 10.1074/jbc.M006545200 11035013

[B150] Ouimet M. Ediriweera H. Afonso M. S. Ramkhelawon B. Singaravelu R. Liao X. (2017). microRNA-33 Regulates Macrophage Autophagy in Atherosclerosis. Arterioscler Thromb. Vasc. Biol. 37 (6), 1058–1067. 10.1161/atvbaha.116.308916 28428217 PMC5494696

[B151] Oumouna-Benachour K. Hans C. P. Suzuki Y. Naura A. Datta R. Belmadani S. (2007). Poly(ADP-ribose) Polymerase Inhibition Reduces Atherosclerotic Plaque Size and Promotes Factors of Plaque Stability in Apolipoprotein E-Deficient Mice: Effects on Macrophage Recruitment, Nuclear Factor-kappaB Nuclear Translocation, and Foam Cell Death. Circulation 115 (18), 2442–2450. 10.1161/circulationaha.106.668756 17438151

[B152] Panousis C. G. Evans G. Zuckerman S. H. (2001). TGF-beta Increases Cholesterol Efflux and ABC-1 Expression in Macrophage-Derived Foam Cells: Opposing the Effects of IFN-Gamma. J. Lipid Res. 42 (5), 856–863. 10.1016/s0022-2275(20)31648-5 11352993

[B153] Park H. J. Lee S. J. Kim S. H. Han J. Bae J. Kim S. J. (2011). IL-10 Inhibits the Starvation Induced Autophagy in Macrophages via Class I Phosphatidylinositol 3-kinase (PI3K) Pathway. Mol. Immunol. 48 (4), 720–727. 10.1016/j.molimm.2010.10.020 21095008

[B154] Park S. H. (2021). Regulation of Macrophage Activation and Differentiation in Atherosclerosis. J. Lipid Atheroscler. 10, 251–267. 10.12997/jla.2021.10.3.251 34621697 PMC8473962

[B155] Park Y. M. Drazba J. A. Vasanji A. Egelhoff T. Febbraio M. Silverstein R. L. (2012). Oxidized LDL/CD36 Interaction Induces Loss of Cell Polarity and Inhibits Macrophage Locomotion. Mol. Biol. Cel 23 (16), 3057–3068. 10.1091/mbc.E11-12-1051 PMC3418302 22718904

[B156] Pham L. M. Kim E. C. Ou W. Phung C. D. Nguyen T. T. Pham T. T. (2021). Targeting and Clearance of Senescent Foamy Macrophages and Senescent Endothelial Cells by Antibody-Functionalized Mesoporous Silica Nanoparticles for Alleviating Aorta Atherosclerosis. Biomaterials 269, 120677. 10.1016/j.biomaterials.2021.120677 33503557

[B157] Phillips M. C. (2018). Is ABCA1 a Lipid Transfer Protein? J. Lipid Res. 59 (5), 749–763. 10.1194/jlr.R082313 29305383 PMC5928442

[B158] Pinderski L. J. Fischbein M. P. Subbanagounder G. Fishbein M. C. Kubo N. Cheroutre H. (2002). Overexpression of Interleukin-10 by Activated T Lymphocytes Inhibits Atherosclerosis in LDL Receptor-Deficient Mice by Altering Lymphocyte and Macrophage Phenotypes. Circ. Res. 90 (10), 1064–1071. 10.1161/01.res.0000018941.10726.fa 12039795

[B159] Porsch F. Kiss M. G. Goederle L. Hendrikx T. Hladik A. Knapp S. (2020). Haematopoetic TREM2 Deficiency Modulates Atherosclerosis and Lipid Metabolism. Atherosclerosis 315, e58–e59. 10.1016/j.atherosclerosis.2020.10.180

[B160] Poznyak A. V. Nikiforov N. G. Markin A. M. Kashirskikh D. A. Myasoedova V. A. Gerasimova E. V. (2021). Overview of OxLDL and its Impact on Cardiovascular Health: Focus on Atherosclerosis. Front. Pharmacol. 11, 2248. 10.3389/fphar.2020.613780 PMC7836017 33510639

[B161] Prattichizzo F. Bonafè M. Olivieri F. Franceschi C. (2016). Senescence Associated Macrophages and "Macroph-Aging": Are They Pieces of the Same Puzzle? Aging (Albany NY) 8 (12), 3159–3160. 10.18632/aging.101133 27941213 PMC5270660

[B162] Que X. Hung M. Y. Yeang C. Gonen A. Prohaska T. A. Sun X. (2018). Oxidized Phospholipids Are Proinflammatory and Proatherogenic in Hypercholesterolaemic Mice. Nature 558 (7709), 301–306. 10.1038/s41586-018-0198-8 29875409 PMC6033669

[B163] Quiding-Järbrink M. Raghavan S. Sundquist M. (2010). Enhanced M1 Macrophage Polarization in Human helicobacter Pylori-Associated Atrophic Gastritis and in Vaccinated Mice. PLoS One 5 (11), e15018. 10.1371/journal.pone.0015018 21124899 PMC2990716

[B164] Raggi F. Pelassa S. Pierobon D. Penco F. Gattorno M. Novelli F. (2017). Regulation of Human Macrophage M1-M2 Polarization Balance by Hypoxia and the Triggering Receptor Expressed on Myeloid Cells-1. Front. Immunol. 8, 1097. 10.3389/fimmu.2017.01097 28936211 PMC5594076

[B165] Reiss A. B. Siegart N. M. De Leon J. (2017). Interleukin-6 in Atherosclerosis: Atherogenic or Atheroprotective? Clin. Lipidol. 12 (1), 14–23.

[B166] Remmerie A. Scott C. L. (2018). Macrophages and Lipid Metabolism. Cell Immunol 330, 27–42. 10.1016/j.cellimm.2018.01.020 29429624 PMC6108423

[B167] Ricci R. Sumara G. Sumara I. Rozenberg I. Kurrer M. Akhmedov A. (2004). Requirement of JNK2 for Scavenger Receptor A-Mediated Foam Cell Formation in Atherogenesis. Science 306 (5701), 1558–1561. 10.1126/science.1101909 15567863

[B168] Ridker P. M. Libby P. MacFadyen J. G. Thuren T. Ballantyne C. Fonseca F. (2018). Modulation of the Interleukin-6 Signalling Pathway and Incidence Rates of Atherosclerotic Events and All-Cause Mortality: Analyses from the Canakinumab Anti-inflammatory Thrombosis Outcomes Study (CANTOS). Eur. Heart J. 39 (38), 3499–3507. 10.1093/eurheartj/ehy310 30165610

[B169] Ridker P. M. Thuren T. Zalewski A. Libby P. (2011). Interleukin-1β Inhibition and the Prevention of Recurrent Cardiovascular Events: Rationale and Design of the Canakinumab Anti-inflammatory Thrombosis Outcomes Study (CANTOS). Am. Heart J. 162 (4), 597–605. 10.1016/j.ahj.2011.06.012 21982649

[B170] Ridker P. M. Devalaraja M. Baeres F. M. M. Engelmann M. D. M. Hovingh G. K. Ivkovic M. (2021). IL-6 Inhibition with Ziltivekimab in Patients at High Atherosclerotic Risk (RESCUE): a Double-Blind, Randomised, Placebo-Controlled, Phase 2 Trial. The Lancet 397 (10289), 2060–2069. 10.1016/s0140-6736(21)00520-1 34015342

[B171] Robbins C. S. Hilgendorf I. Weber G. F. Theurl I. Iwamoto Y. Figueiredo J. L. (2013a). Local Proliferation Dominates Lesional Macrophage Accumulation in Atherosclerosis. Nat. Med. 19 (9), 1166–1172. 10.1038/nm.3258 23933982 PMC3769444

[B172] Robbins C. S. Hilgendorf I. Weber G. F. Theurl I. Iwamoto Y. Figueiredo J. L. (2013b). Local Proliferation Dominates Lesional Macrophage Accumulation in Atherosclerosis. Nat. Med. 19 (9), 1166–72. 10.1038/nm.3258 23933982 PMC3769444

[B173] Robertson A. K. Hansson G. K. (2006). T Cells in Atherogenesis: for Better or for Worse? Arterioscler Thromb. Vasc. Biol. 26 (11), 2421–2432. 10.1161/01.ATV.0000245830.29764.84 16973967

[B174] Robinson N. Ganesan R. Hegedűs C. Kovács K. Kufer T. A. Virág L. (2019). Programmed Necrotic Cell Death of Macrophages: Focus on Pyroptosis, Necroptosis, and Parthanatos. Redox Biol. 26, 101239. 10.1016/j.redox.2019.101239 31212216 PMC6582207

[B175] Rogers C. Fernandes-Alnemri T. Mayes L. Alnemri D. Cingolani G. Alnemri E. S. (2017). Cleavage of DFNA5 by Caspase-3 during Apoptosis Mediates Progression to Secondary Necrotic/pyroptotic Cell Death. Nat. Commun. 8, 14128. 10.1038/ncomms14128 28045099 PMC5216131

[B176] Romuk E. Skrzep-Poloczek B. Wojciechowska C. Tomasik A. Birkner E. Wodniecki J. (2002). Selectin-P and Interleukin-8 Plasma Levels in Coronary Heart Disease Patients. Eur. J. Clin. Invest. 32 (9), 657–661. 10.1046/j.1365-2362.2002.01053.x 12486864

[B177] Rong J. X. Shapiro M. Trogan E. Fisher E. A. (2003). Transdifferentiation of Mouse Aortic Smooth Muscle Cells to a Macrophage-like State after Cholesterol Loading. Proc. Natl. Acad. Sci. U S A. 100 (23), 13531–13536. 10.1073/pnas.1735526100 14581613 PMC263848

[B178] Sano S. Oshima K. Wang Y. Katanasaka Y. Sano M. Walsh K. (2018). CRISPR-mediated Gene Editing to Assess the Roles of Tet2 and Dnmt3a in Clonal Hematopoiesis and Cardiovascular Disease. Circ. Res. 123 (3), 335–341. 10.1161/CIRCRESAHA.118.313225 29728415 PMC6054544

[B179] Schiopu A. Frendéus B. Jansson B. Söderberg I. Ljungcrantz I. Araya Z. (2007). Recombinant Antibodies to an Oxidized Low-Density Lipoprotein Epitope Induce Rapid Regression of Atherosclerosis in Apobec-1(-/-)/low-Density Lipoprotein Receptor(-/-) Mice. J. Am. Coll. Cardiol. 50 (24), 2313–2318. 10.1016/j.jacc.2007.07.081 18068040

[B180] Scull C. M. Hays W. D. Fischer T. H. (2010). Macrophage Pro-inflammatory Cytokine Secretion Is Enhanced Following Interaction with Autologous Platelets. J. Inflamm. (Lond) 7, 53. 10.1186/1476-9255-7-53 21067617 PMC2988777

[B181] Shami A. Edsfeldt A. Shore A. C. Natali A. Khan F. Nilsson J. (2020). CD40 Levels in Plasma Are Associated with Cardiovascular Disease and in Carotid Plaques with a Vulnerable Plaque Phenotype and Remodelling. Eur. Heart J. 41 (Suppl. ment_2), ehaa946–3782. 10.1093/ehjci/ehaa946.3782 PMC8521258 34649381

[B182] Sinha S. K. Miikeda A. Fouladian Z. Mehrabian M. Edillor C. Shih D. (2021). Local M-CSF (Macrophage colony-stimulating Factor) Expression Regulates Macrophage Proliferation and Apoptosis in Atherosclerosis. Arteriosclerosis, Thromb. Vasc. Biol. 41 (1), 220–233. 10.1161/ATVBAHA.120.315255 PMC7769919 33086870

[B183] Skuratovskaia D. Vulf M. Khaziakhmatova O. Malashchenko V. Komar A. Shunkin E. (2020). Tissue-Specific Role of Macrophages in Noninfectious Inflammatory Disorders. Biomedicines 8 (10), 400. 10.3390/biomedicines8100400 PMC7600904 33050138

[B184] Smith M. S. Bentz G. L. Smith P. M. Bivins E. R. Yurochko A. D. (2004). HCMV Activates PI(3)K in Monocytes and Promotes Monocyte Motility and Transendothelial Migration in a PI(3)K-dependent Manner. J. Leukoc. Biol. 76 (1), 65–76. 10.1189/jlb.1203621 15107461

[B185] Sodhi N. Brown D. L. (2018). Pathophysiology of Acute Coronary Syndromes: Plaque Rupture and Atherothrombosis. Philadelphia: Cardiac Intensive Care-E-Book, 68.

[B186] Spiller K. L. Anfang R. R. Spiller K. J. Ng J. Nakazawa K. R. Daulton J. W. (2014). The Role of Macrophage Phenotype in Vascularization of Tissue Engineering Scaffolds. Biomaterials 35 (15), 4477–4488. 10.1016/j.biomaterials.2014.02.012 24589361 PMC4000280

[B187] Subedi L. Lee S. E. Madiha S. Gaire B. P. Jin M. Yumnam S. (2020). Phytochemicals against TNFα-Mediated Neuroinflammatory Diseases. Int. J. Mol. Sci. 21 (3), 764. 10.3390/ijms21030764 PMC7037901 31991572

[B188] Swirski F. K. Nahrendorf M. (2013a). Leukocyte Behavior in Atherosclerosis, Myocardial Infarction, and Heart Failure. Science 339 (6116), 161–166. 10.1126/science.1230719 23307733 PMC3891792

[B189] Syväranta S. Alanne-Kinnunen M. Oörni K. Oksjoki R. Kupari M. Kovanen P. T. (2014). Potential Pathological Roles for Oxidized Low-Density Lipoprotein and Scavenger Receptors SR-AI, CD36, and LOX-1 in Aortic Valve Stenosis. Atherosclerosis 235 (2), 398–407. 10.1016/j.atherosclerosis.2014.05.933 24929820

[B190] Tabas I. (2010). The Role of Endoplasmic Reticulum Stress in the Progression of Atherosclerosis. Circ. Res. 107 (7), 839–850. 10.1161/circresaha.110.224766 20884885 PMC2951143

[B191] Tacke F. Alvarez D. Kaplan T. J. Jakubzick C. Spanbroek R. Llodra J. (2007). Monocyte Subsets Differentially Employ CCR2, CCR5, and CX3CR1 to Accumulate within Atherosclerotic Plaques. J. Clin. Invest. 117 (1), 185–194. 10.1172/JCI28549 17200718 PMC1716202

[B192] Tang J. Lobatto M. E. Hassing L. Van Der Staay S. Van Rijs S. M. Calcagno C. (2015). Inhibiting Macrophage Proliferation Suppresses Atherosclerotic Plaque Inflammation. Sci. Adv. 1 (3), e1400223. 10.1126/sciadv.1400223 26295063 PMC4539616

[B193] Tao H. Huang J. Yancey P. G. Yermalitsky V. Blakemore J. L. Zhang Y. (2020). Scavenging of Reactive Dicarbonyls with 2-hydroxybenzylamine Reduces Atherosclerosis in Hypercholesterolemic Ldlr-/- Mice. Nat. Commun. 11 (1), 4084–4115. 10.1038/s41467-020-17915-w 32796843 PMC7429830

[B194] Tao H. Yancey P. G. Babaev V. R. Blakemore J. L. Zhang Y. Ding L. (2015). Macrophage SR-BI Mediates Efferocytosis via Src/PI3K/Rac1 Signaling and Reduces Atherosclerotic Lesion Necrosis. J. Lipid Res. 56 (8), 1449–1460. 10.1194/jlr.M056689 26059978 PMC4513986

[B195] Teh Y. C. Ding J. L. Ng L. G. Chong S. Z. (2019). Capturing the Fantastic Voyage of Monocytes through Time and Space. Front. Immunol. 10, 834. 10.3389/fimmu.2019.00834 31040854 PMC6476989

[B196] Tenger C. Sundborger A. Jawien J. Zhou X. (2005). IL-18 Accelerates Atherosclerosis Accompanied by Elevation of IFN-Gamma and CXCL16 Expression Independently of T Cells. Arterioscler Thromb. Vasc. Biol. 25 (4), 791–796. 10.1161/01.atv.0000153516.02782.65 15604417

[B197] Thayse K. Kindt N. Laurent S. Carlier S. (2020). VCAM-1 Target in Non-invasive Imaging for the Detection of Atherosclerotic Plaques. Biology (Basel) 9 (11), 368. 10.3390/biology9110368 PMC7692297 33138124

[B198] Tian F. Yao J. Yan M. Sun X. Wang W. Gao W. (2016). 5-Aminolevulinic Acid-Mediated Sonodynamic Therapy Inhibits RIPK1/RIPK3-dependent Necroptosis in THP-1-Derived Foam Cells. Sci. Rep. 6 (1), 21992–22013. 10.1038/srep21992 26911899 PMC4766406

[B199] Tyrrell D. J. Goldstein D. R. (2020). Ageing and Atherosclerosis: Vascular Intrinsic and Extrinsic Factors and Potential Role of IL-6. Nat. Rev. Cardiol. 18, 1–11. 10.1038/s41569-020-0431-7 PMC7484613 32918047

[B200] Van Eck M. Twisk J. Hoekstra M. Van Rij B. T. Van der Lans C. A. Bos I. S. (2003). Differential Effects of Scavenger Receptor BI Deficiency on Lipid Metabolism in Cells of the Arterial wall and in the Liver. J. Biol. Chem. 278 (26), 23699–23705. 10.1074/jbc.M211233200 12639961

[B201] Veerman K. M. Williams M. J. Uchimura K. Singer M. S. Merzaban J. S. Naus S. (2007). Interaction of the Selectin Ligand PSGL-1 with Chemokines CCL21 and CCL19 Facilitates Efficient Homing of T Cells to Secondary Lymphoid Organs. Nat. Immunol. 8 (5), 532–539. 10.1038/ni1456 17401367

[B202] Vijayvergiya R. Vadivelu R. (2015). Role of *Helicobacter pylori* Infection in Pathogenesis of Atherosclerosis. World J. Cardiol. 7 (3), 134–143. 10.4330/wjc.v7.i3.134 25810813 PMC4365310

[B203] Virmani R. Burke A. P. Kolodgie F. D. Farb A. (2002). Vulnerable Plaque: the Pathology of Unstable Coronary Lesions. J. Interv. Cardiol. 15 (6), 439–446. 10.1111/j.1540-8183.2002.tb01087.x 12476646

[B204] Wainstein M. V. Mossmann M. Araujo G. N. Gonçalves S. C. Gravina G. L. Sangalli M. (2017). Elevated Serum Interleukin-6 Is Predictive of Coronary Artery Disease in Intermediate Risk Overweight Patients Referred for Coronary Angiography. Diabetol. Metab. Syndr. 9, 67. 10.1186/s13098-017-0266-5 28878828 PMC5585915

[B205] Wang L. Li H. Tang Y. Yao P. (2020). Potential Mechanisms and Effects of Efferocytosis in Atherosclerosis. Front. Endocrinol. (Lausanne) 11, 585285. 10.3389/fendo.2020.585285 33597922 PMC7883484

[B206] Wang N. Liang H. Zen K. (2014a). Molecular Mechanisms that Influence the Macrophage M1-M2 Polarization Balance. Front. Immunol. 5, 614. 10.3389/fimmu.2014.00614 25506346 PMC4246889

[B207] Wang Y. Gao W. Shi X. Ding J. Liu W. He H. (2017). Chemotherapy Drugs Induce Pyroptosis through Caspase-3 Cleavage of a Gasdermin. Nature 547 (7661), 99–103. 10.1038/nature22393 28459430

[B208] Willemsen L. de Winther M. P. (2020). Macrophage Subsets in Atherosclerosis as Defined by Single-Cell Technologies. J. Pathol. 250 (5), 705–714. 10.1002/path.5392 32003464 PMC7217201

[B209] Williams J. W. Winkels H. Durant C. P. Zaitsev K. Ghosheh Y. Ley K. (2020). Single Cell RNA Sequencing in Atherosclerosis Research. Circ. Res. 126 (9), 1112–1126. 10.1161/CIRCRESAHA.119.315940 32324494 PMC7185048

[B210] Winkels H. Ehinger E. Vassallo M. Buscher K. Dinh H. Q. Kobiyama K. (2018). Atlas of the Immune Cell Repertoire in Mouse Atherosclerosis Defined by Single-Cell RNA-Sequencing and Mass Cytometry. Circ. Res. 122 (12), 1675–1688. 10.1161/CIRCRESAHA.117.312513 29545366 PMC5993603

[B211] Wynn T. A. Vannella K. M. (2016). Macrophages in Tissue Repair, Regeneration, and Fibrosis. Immunity 44 (3), 450–462. 10.1016/j.immuni.2016.02.015 26982353 PMC4794754

[B212] Xiong D. Wang Y. You M. (2020). A Gene Expression Signature of TREM2hi Macrophages and γδ T Cells Predicts Immunotherapy Response. Nat. Commun. 11 (1), 5084–5112. 10.1038/s41467-020-18546-x 33033253 PMC7545100

[B213] Xu H. Jiang J. Chen W. Li W. Chen Z. (2019). Vascular Macrophages in Atherosclerosis. J. Immunol. Res. 2019, 4354786. 10.1155/2019/4354786 31886303 PMC6914912

[B214] Xu S. Bai P. Little P. J. Liu P. (2014). Poly(ADP-ribose) Polymerase 1 (PARP1) in Atherosclerosis: from Molecular Mechanisms to Therapeutic Implications. Med. Res. Rev. 34 (3), 644–675. 10.1002/med.21300 24002940

[B215] Xu S. Ogura S. Chen J. Little P. J. Moss J. Liu P. (2013). LOX-1 in Atherosclerosis: Biological Functions and Pharmacological Modifiers. Cel Mol Life Sci 70 (16), 2859–2872. 10.1007/s00018-012-1194-z PMC4142049 23124189

[B216] Yamamoto S. Yancey P. G. Zuo Y. Ma L. J. Kaseda R. Fogo A. B. (2011). Macrophage Polarization by Angiotensin II-type 1 Receptor Aggravates Renal Injury-Acceleration of Atherosclerosis. Arterioscler Thromb. Vasc. Biol. 31 (12), 2856–2864. 10.1161/atvbaha.111.237198 21979434 PMC3227118

[B217] Yan G. Elbadawi M. Efferth T. (2020). Multiple Cell Death Modalities and Their Key Features. World Acad. Sci. J. 2 (2), 39–48.

[B218] Yang S. Li J. Chen Y. Zhang S. Feng C. Hou Z. (2019). MicroRNA-216a Promotes M1 Macrophages Polarization and Atherosclerosis Progression by Activating Telomerase via the Smad3/NF-Κb Pathway. Biochim. Biophys. Acta Mol. Basis Dis. 1865 (7), 1772–1781. 10.1016/j.bbadis.2018.06.016 29940270

[B219] Yin Q. Chang H. Shen Q. Xing D. (2021). Photobiomodulation Therapy Promotes the ATP-Binding Cassette Transporter A1-dependent Cholesterol Efflux in Macrophage to Ameliorate Atherosclerosis. J. Cel Mol Med 25 (11), 5238–5249. 10.1111/jcmm.16531 PMC8178257 33951300

[B220] Yu X. H. Fu Y. C. Zhang D. W. Yin K. Tang C. K. (2013). Foam Cells in Atherosclerosis. Clin. Chim. Acta 424, 245–252. 10.1016/j.cca.2013.06.006 23782937

[B221] Zeng C. Wang R. Tan H. (2019). Role of Pyroptosis in Cardiovascular Diseases and its Therapeutic Implications. Int. J. Biol. Sci. 15 (7), 1345–1357. 10.7150/ijbs.33568 31337966 PMC6643148

[B222] Zernecke A. Winkels H. Cochain C. Williams J. W. Wolf D. Soehnlein O. (2020). Meta-analysis of Leukocyte Diversity in Atherosclerotic Mouse Aortas. Circ. Res. 127 (3), 402–426. 10.1161/CIRCRESAHA.120.316903 32673538 PMC7371244

[B223] Zhang H. Park Y. Wu J. Chen Xp. Lee S. Yang J. (2009). Role of TNF-Alpha in Vascular Dysfunction. Clin. Sci. (Lond) 116 (3), 219–230. 10.1042/cs20080196 19118493 PMC2620341

[B224] Zhang X. Niessner A. Nakajima T. Ma-Krupa W. Kopecky S. L. Frye R. L. (2006). Interleukin 12 Induces T-Cell Recruitment into the Atherosclerotic Plaque. Circ. Res. 98 (4), 524–531. 10.1161/01.RES.0000204452.46568.57 16424368

[B225] Zhang Y. Wang Y. Zhou D. Zhang L. S. Deng F. X. Shu S. (2019). Angiotensin II Deteriorates Advanced Atherosclerosis by Promoting MerTK Cleavage and Impairing Efferocytosis through the AT1R/ROS/p38 MAPK/ADAM17 Pathway. Am. J. Physiol. Cel Physiol 317 (4), C776–C787. 10.1152/ajpcell.00145.2019 31390228

[B226] Zhang Y. Shi X. Han J. Peng W. Fang Z. Zhou Y. (2021). Convallatoxin Promotes M2 Macrophage Polarization to Attenuate Atherosclerosis through PPARγ-Integrin αvβ5 Signaling Pathway. Dddt 15, 803–812. 10.2147/dddt.s288728 33654384 PMC7914072

[B227] Zhu X. D. Zhuang Y. Ben J. J. Qian L. L. Huang H. P. Bai H. (2011). Caveolae-dependent Endocytosis Is Required for Class A Macrophage Scavenger Receptor-Mediated Apoptosis in Macrophages. J. Biol. Chem. 286 (10), 8231–8239. 10.1074/jbc.M110.145888 21205827 PMC3048709

[B228] Zuo S. Yu J. Pan H. Lu L. (2020). Novel Insights on Targeting Ferroptosis in Cancer Therapy. Biomark Res. 8 (1), 50–11. 10.1186/s40364-020-00229-w 33024562 PMC7532638

